# The de novo genome assembly of *Tapiscia sinensis* and the transcriptomic and developmental bases of androdioecy

**DOI:** 10.1038/s41438-020-00414-w

**Published:** 2020-12-01

**Authors:** Peng Zhao, Guiliang Xin, Feng Yan, Huan Wang, Xiaolong Ren, Keith Woeste, Wenzhe Liu

**Affiliations:** 1 Key Laboratory of Resource Biology and Biotechnology in Western China, Ministry of Education, College of Life Sciences, Northwest University, Xi’an, Shaanxi 710069 China; 2 USDA Forest Service Hardwood Tree Improvement and Regeneration Center (HTIRC), Department of Forestry and Natural Resources, Purdue University, 715 West State Street, West Lafayette, IN 47907 USA

**Keywords:** Population genetics, Plant physiology, Plant development

## Abstract

*Tapiscia sinensis* (Tapisciaceae) possesses an unusual androdioecious breeding system that has attracted considerable interest from evolutionary biologists. Key aspects of *T. sinensis* biology, including its biogeography, genomics, and sex-linked genes, are unknown. Here, we report the first de novo assembly of the genome of *T. sinensis*. The genome size was 410 Mb, with 22,251 predicted genes. Based on whole-genome resequencing of 55 trees from 10 locations, an analysis of population genetic structure indicated that *T. sinensis* has fragmented into five lineages, with low intrapopulation genetic diversity and little gene flow among populations. By comparing whole-genome scans of male versus hermaphroditic pools, we identified 303 candidate sex-linked genes, 79 of which (25.9%) were located on scaffold 25. A 24-kb region was absent in hermaphroditic individuals, and five genes in that region, *TsF-box4*, *TsF-box10*, *TsF-box13*, *TsSUT1*, and *TsSUT4*, showed expression differences between mature male and hermaphroditic flowers. The results of this study shed light on the breeding system evolution and conservation genetics of the Tapisciaceae.

## Introduction

*Tapiscia sinensis* Oliv. (Tapisciaceae), is a woody, perennial, androdioecious species^1–6^. Androdioecy is a rare breeding system in which populations consist of only male and hermaphroditic individuals^7,8^. Hermaphroditic individuals have both male and female functions^9^. Functional androdioecy is found in less than 0.005% of angiosperms^10^, including *Mercurialis annua*^11^, *Datisca glomerata*^11,12^, *Schizopepon bryoniaefolius*^9^, *Laguncularia racemosa*^13^, and *Osmanthus fragrans*^14,15^. *T. sinensis* has research value as a model for the study of the evolution and maintenance of androdioecy^1,2,4^. In *T. sinensis*, floral buds on hermaphroditic individuals initiate differentiation in May, flower in late June, and are fertilized in early July^1,2^. After 9 months of quiescence, young fruits resume development in April, reaching maturity in September. Thus, the flower and fruit development of *T*. *sinensis* lasts 17 months^1^, a phenomenon more common in gymnosperms^16,17^. Other angiosperms with long flower and fruit development^18,19^ include *Carpinus turczaninowii*, *Ostryopsis davidiana*^18^, *Betula platyphylla*^19^, and trees in section *Lobatae* of *Quercus*.

**Fig. 1:**
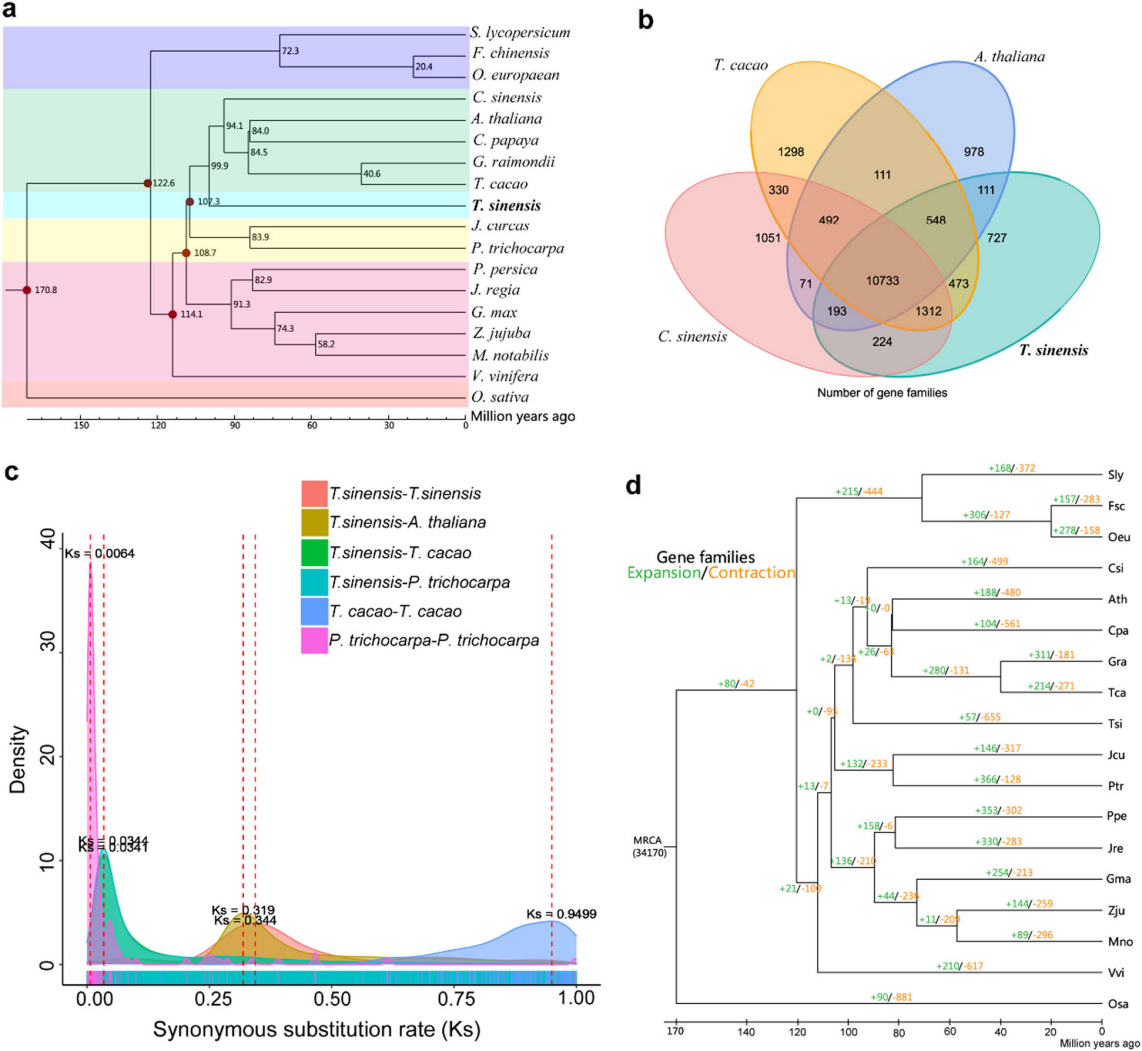
Comparative analysis of the *Tapiscia sinensis* genome with the genomes of other plant species. **a** Phylogenetic analysis of *T. sinensis* and other sequenced plants. *O. sativa* was used as an outgroup. **b** Venn diagram of shared orthologous gene families in *T. sinensis*, *A. thaliana*, *T. cacao*, and *C. sinensis*. **c** Density distributions of the synonymous substitution rate (Ks) between duplicated genes of syntenic regions in *A. thaliana*, *P. trichocarpa*, *T. cacao*, and *T. sinensis* are shown by different colors. **d** Phylogenetic tree and expansion and contraction of gene families. The phylogenetic tree was constructed from a concatenated alignment of 509 families of single-copy genes from 18 higher plant species. Sly, *S. lycopersicum*; Fsc, *F. chinensis*; Oue*, O. europaea;* Csi *C. sinensis*; Ath*, A. thaliana;* Cpa*, C. papaya;* Gra*, G. raimondii*; Tca*, T. cacao;* Tsi, *T. sinensis;* Jcu*, J. curcas*; Ptr, *P. trichocarpa*; Gma*, G. max*; Jre, *J. regia*; Ppe*, P. persica*; Zju*, Z. jujube*; Mno, *M. notabilis;* Vvi, *V. vinifera*; Osa, *O._sativa* (details in Supplementary Table 11). MRCA is the most recent common ancestor. Gene family expansions are indicated in green, and gene family contractions are indicated in orange

*Tapiscia* was formerly widespread in the Northern Hemisphere. More than ten fossil species are described among the Eocene flora (~60 million years ago, Ma) of China^20^, England^21^, Germany^21^, Oregon (USA)^22^, and Canada^23^ (Fig. 1a). Currently, the genus comprises a single species, *T. sinensis*, which is distributed in central, subtropical mountains of China, south of the Yangtze River between elevations of 250 and 2200 m, and in northern Vietnam^1–5,24,25^. Due to deforestation and reclamation, natural populations of *T. sinensis* are rare [International Union for Conservation of Nature (IUCN) Red List]; it grows only in small, scattered, disjunct sites, mostly at higher elevations^25–28^. The phylogenetic position of *T. sinensis* makes it an important species for understanding angiosperm evolution. In addition, this tree species has great value in Chinese traditional medicine and in landscape horticulture^2,25^.

Here, we report the sequencing and de novo assembly of the genome of a *T. sinensis* individual growing in the Qinling Mountains, Shaanxi Province. We used the annotated genome to determine the number of protein-coding genes in *Tapiscia*; to evaluate its genome structure, including whole-genome duplication; to evaluate its phylogenetic position; and to estimate the timing of its divergence from its most recent ancestor. An important objective of this research was to identify regions of the *Tapiscia* genome linked to sex determination and to identify and describe the expression of genes that contribute to *Tapiscia’s* unusual reproductive habit.

## Results

### Genome sequencing, assembly, repeat sequence annotation, and gene prediction

We sequenced a single genotype of *T. sinensis*; the source tree was a wild male individual from the Qinling Mountains, China. The input data for the whole-genome assembly included three libraries with insert sizes from 350 bp to 20 kb (Supplementary Table 1). Most of the data were sequences from the ends of short (400 bp) fragments; 59.30 Gb were sequenced from an Illumina paired-end library with an insert size of 350 bp. The sequence data represented ~131.97× coverage of the genome (Supplementary Table 1). We generated longer-fragment libraries of 20 kb that assisted greatly in linking contigs into scaffolds using the PacBio Sequel platform. Using the PacBio platform, we generated 39.96 Gb of sequence data representing approximately 88.93× coverage of the genome. We also sequenced 54.93 Gb of data using the 10X Genomics platform, which generates 500–700 bp read lengths. These data represented ~122.25× coverage of the genome. The final genome assembly was 410 Mb, with contig and scaffold N50 sizes of 1.72 and 4.64 kb, respectively (Table 1 and Supplementary Tables 2, 3). The final assembly size was ~96% of the estimated total genome size based on 17-mer analysis (427.77 Mb) (Supplementary Figs. 1, 2). A total of 154.19 Gb of sequence resulted in ~343.15× coverage of the genome. We identified 2267 complete benchmarking universal single-copy orthologs (BUSCOs), including 2153 single-copy BUSCOs, 113 duplicated BUSCOs, 20 fragmented BUSCOs, and 37 missing BUSCOs, in the assembled *Tapiscia sinensis* genome (Table 1). The chloroplast genome of *T. sinensis* was assembled separately into one circular genome with a size of 161,100 bp, including a large single-copy (LSC) region of 87,766 bp, a small single-copy (SSC) region of 18,520 bp, and an inverted repeat region (IRa and IRb) of ~27,407 bp^27^. The tandem repeat (long terminal repeat, LTR) sequence annotations showed that transposable elements accounted for ~5.2% of the reference genome (Table 1 and Supplementary Table 2). The GC content was ~33.41% across the *T. sinensis* genome.

**Table 1:** Statistics and BUSCO evaluation of the *Tapiscia sinensis* genome

Estimate of genome size	410 Mb
GC content	33.41%
N50 length (contig)	1757 bp
Number of contigs	836,436
N50 length (scaffold)	4747 bp
Number of scaffolds	557
Number of genes	22,251
Average gene length	4990 bp
Average coding sequence length	1210 bp
Average exon length	302 bp
Average TE protein length	278 bp
Tandem repeat	228,544 bp
Complete BUSCOs	2267 (97.5%)
Complete and single-copy BUSCOs	2153 (92.6%)
Complete and duplicated BUSCOs	113 (4.9%)
Fragmented BUSCOs	20 (0.9%)
Missing BUSCOs	37 (1.6%)
Total BUSCO groups searched	2326 (100%)

### Transcriptome sequencing and assembly

We generated a total of 29.7 Gb of RNA sequence data from male flowers, hermaphroditic flowers, fruits, roots, bark, leaves, young stems (tender shoots), and mature stems to annotate the genome (Supplementary Table 4). Trinity splicing results displayed 147,609 subclusters in PASA software by alignment clustering. The transcriptome sequences covered 84.91% of the reference genome. Over 456 million paired-end reads were sequenced across the six tissues. As a measure of the quality of the genome assembly of *T. sinensis*, 77.34% of 197,283 unigenes from the RNA-seq data were aligned to the genome (Supplementary Table 5). More than 80% of transcript reads could be mapped to the Illumina reads, with only slight variation based on tissue source [fruit (88.51%), root (88.10%), bark (88.42%), leaf (80.52%), young stem (90.50%), and mature stem (88.07%)] (Supplementary Tables 4, 5). We defined 22,251 protein-coding genes (Table 1 and Supplementary Tables 2, 3, 6). Homology-based gene searches and searches for predicted noncoding RNA (ncRNA) (Supplementary Table 2) yielded 1180 ribosomal RNA (rRNA) genes, 497 transfer RNA (tRNA) genes, 383 small nuclear RNA (snRNA) genes, and 364 microRNA (miRNA) genes (Supplementary Table 2).

A total of 92,073 simple sequence repeats (SSRs) were found in *T. sinensis* (Supplementary Tables 7–9). We annotated 72,706 SSRs for studying the population genetics of *T. sinensis* (Supplementary Tables 7–9). We designed 150 pairs of primers and found 68 polymorphic loci (45.5%). Six polymorphic SSRs were used for genetic analysis of 51 *T. sinensis* individuals^28^. We used the sex-linked SSR locus TS095 (forward: TTGTCCCTCTCAACTTCGCT, reverse: AAAATCAACCAGCCAGTTCG)^28^ to evaluate 1041 *T. sinensis* offspring (seeds) from five locations, including the Northwest University campus, Qinling Mountains, Wuling Mountains, Daba Mountains, and Luoxiao Mountains (Supplementary Table 10). The results based on segregation of the TS095 SSR alleles showed that the sex ratio among these 1041 offspring was nearly 1:1; the numbers of males and hermaphrodites were 505 and 536, respectively (Supplementary Table 10).

### Comparative analysis of the *T. sinensis* genome and gene-based phylogeny

The annotated gene models (*N* = 22,251) were grouped into gene families (*N* = 6,767) comprising at least two genes based on sequence similarity. We identified 229 *T. sinensis*-specific genes that did not cluster with any genes from any of 17 other plant species: *Morus notabilis, Ziziphus jujuba* (jujube), *Prunus persica* (peach), *Oryza sativa* (rice), *Arabidopsis thaliana, Juglans regia* (common walnut), *Vitis vinifera* (grape), *Jatropha curcas*, *Glycine max* (soybean), *Populus trichocarpa* (poplar), *Gossypium raimondii* (cotton), *Theobroma cacao* (cocoa), *Fraxinus chinensis*, *Carica papaya* (papaya), *Solanum lycopersicum* (tomato), *Olea europaea* and *Citrus sinensis* (orange) (Supplementary Table 11). A total of 10,733 gene families were found based on comparisons among *T. sinensis*, *C. sinensis*, *A. thaliana*, and *T. cacao*; this four-species comparison revealed 727 species-specific genes in *T. sinensis* (Fig. 1b and Supplementary Tables 12, 13). We constructed a phylogenetic tree of *T. sinensis* together with 17 other sequenced plant genomes based on 594 single-copy genes using the monocot rice (*O. sativa*) as an outgroup (Fig. 1a and Supplementary Table 1 and Supplementary Fig. 3). The resulting tree shows that *T. sinensis* diverged approximately 100 Ma from a clade containing the Rutaceae, Brassicaceae, and Malvaceae, among others (Fig. 1a).

The distribution of Ks values peaked at approximately 0.34 in *T. sinensis*, indicating that *T. sinensis* has not undergone any recent lineage-specific whole-genome duplication (WGD) events (Fig. 1c). The distribution of 4DTv values at approximately 0.45 supported this interpretation of the fourfold degenerate site (4DTv) results (Supplementary Fig. 4). Pairwise orthology between species, including the orthology between *T. sinensis* and *P. trichocarpa* (Salicaceae), *T. sinensis* and *T. cacao*, and *T. sinensis* and *A. thaliana*, showed 4DTv distance peaks at ~0.30, ~0.30, and 0.60, respectively, indicating that the divergence time of *T. sinensis* from *A. thaliana* was earlier than that from *P. trichocarpa*, consistent with the phylogenetic tree (Fig. 1a).

To identify gene families that had expanded or contracted only in *T. sinensis*, we compared gene families from *T. sinensis* with those of 17 other representative species and with an ancestral species. We identified a total of 57 gene families that have undergone significant (*p* < 0.01) expansion in the *T. sinensis* genome (Supplementary Table 14). The results of Kyoto Encyclopedia of Genes and Genomes (KEGG) pathway enrichment analysis revealed that these families were enriched with genes involved in phototransduction, anthocyanin biosynthesis, calcium signaling, oocyte meiosis, and plant–pathogen interaction, indicating that these pathways have evolved distinctly in *T. sinensis* compared to other plant species. Information about enrichment provides a basic resource for understanding secondary metabolism in *T. sinensis* (Supplementary Tables 14–16). Analysis of gene ontology (GO) and KEGG pathways showed that male and hermaphroditic plants were enriched in different annotations; for example, in males, we found that the greatest number of enriched genes were related to metabolic process, catalytic activity, and hydrolase activity, whereas enrichment in the hermaphroditic plant genome was in binding genes (Fig. 4b and Supplementary Figs. 5, 6 and Supplementary Table 17).

### Genetic diversity and population structure of *T. sinensis*

**Fig. 2:**
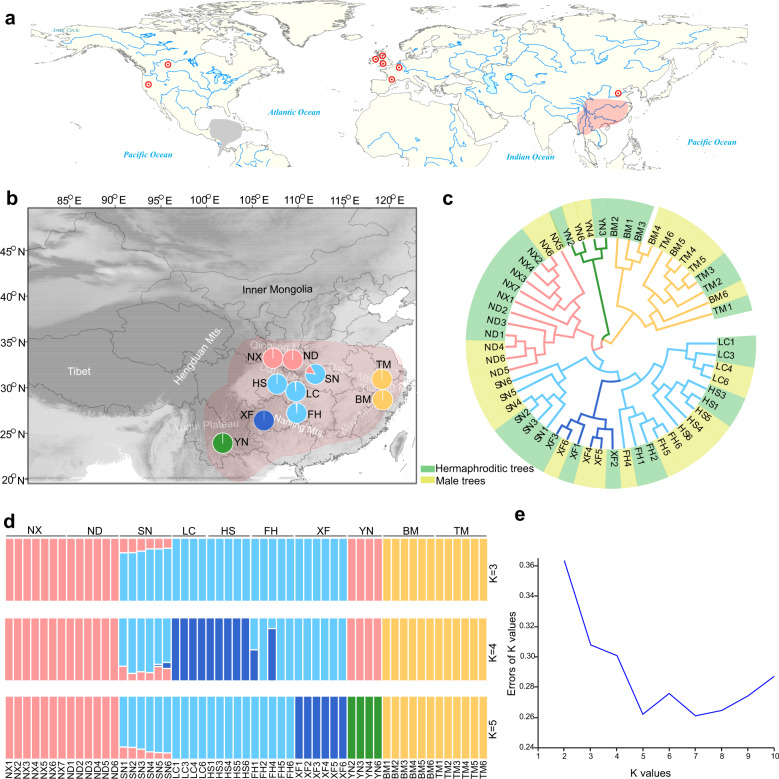
Spatial genetic structure of *Tapiscia* (Tapisciaceae). **a** Locations of fossils of Tapisciaceae (red dots). *Huertea* is from the New World only, and *Tapiscia* is Eurasian. Current distributions of *Huertea* and *Tapiscia* are shown with gray and pink shading, respectively. **b** Spatial genetic structure of *Tapiscia* from ten sampled locations within the species’ natural range; pie chart colors reflect the probability of sample assignment based on independent, nonmissing SNPs analyzed using the software STRUCTURE at the most likely *K* = 5. **c** Neighbor-joining tree based on all SNPs in 55 individuals of *T. sinensis*. Samples shaded with yellow-green were male trees, samples shaded with green were hermaphroditic trees; color-coding of the branches reflects the structure of genetic groups (see panel **d**) at *K* = 5. **d** Results of STRUCTURE analyses of 55 individuals of *T. sinensis* at *K* = 3–5. **e** Plot of cross-validation errors for STRUCTURE runs where *K* values ranged from 2 to 10 (optimal *K* = 5)

To investigate the genomic diversity, population structure, and biogeography of *T. sinensis*, we sequenced 55 trees from 10 locations that represent the species’ entire natural distribution in China (Fig. 2a, b and Supplementary Table 18). Thus, we generated a total of 560 Gb of high-quality, cleaned sequence data, at an average of 10.19 Gb per sample (equivalent to ~25× coverage of the ~410-Mb sequence of the wild male individual “Tree168”). Sequences of Tree 168 were also mapped to the reference genome (mapping rate of 90.04%) (Supplementary Table 19). A total of 11,431,841 single nucleotide polymorphisms (SNPs) were identified among 55 individuals, with an average of ~27 SNPs per kb of the *T. sinensis* genome (Supplementary Table 19). A total of 62.1% of these SNPs were intergenic; 4.2% of the SNPs were located in coding sequences (CDS), 2.12% were synonymous, and 2.06% were nonsynonymous, resulting in a nonsynonymous/synonymous ratio of 0.97 (Supplementary Table 20).

We analyzed the population structure of 55 *T. sinensis* trees using three methods: STRUCTURE runs on genomic SNPs, a neighbor-joining (NJ) tree, and a principal component analysis (PCA) of all SNPs (Fig. 2b–d and Supplementary Fig. 7). The clearest differentiation was found in the nuclear genome; five distinct groups emerged from the analysis. The populations of Zhejiang Province and the Tianmu Mountains (TM and BM, respectively, yellow in Fig. 2b) were the most geographically isolated and most genetically distinct (Fig. 2). The Yunnan Province (YN) population (southwestern China) was the second most genetically distinct. The third group was centered in the Qinling Mountains (NX and ND), the fourth population was from Guizhou Province (XF), and the fifth group of samples constituted a population in Hubei Province [(Shennongjia national forest (SN) and Lichuan County (LC)], Hunan Province (FH), and Chongqing (HS) (Fig. 2 and Supplementary Fig. 7 and Supplementary Table 18).

Bayesian analysis of the genetic structure of the 55 trees from all 10 sampled sites produced populations that corresponded exactly with the clusters identified using neighbor joining (Fig. 2b–d and Supplementary Fig. 7). The underlying hierarchical structure of the *T. sinensis* trees was best represented at *K* = 5, in part because the individuals clustered into populations that corresponded to the geographic regions from which they were sampled (Fig. 2e). The results of PCA revealed five clusters of samples corresponding to the same groups identified by STRUCTURE and further supported the genetic distinctiveness of populations TM and BM (Fig. 2 and Supplementary Fig. 7). The distribution of *T. sinensis* in China is marked by strong fragmentation. The populations of Zhejiang, Yunnan, and Guizhou are highly differentiated genetically from those of other regions, and there was no gene flow signal into these populations from populations in other regions. Two populations of *T. sinensis* growing in the Qinling Mountains clustered into one genetic component. *T. sinensis* trees in the Shennongjia Mountains (SN) population showed evidence of gene introgression from the NX and ND populations (Fig. 2).

### Morphology and development of male and hermaphroditic flowers

**Fig. 3:**
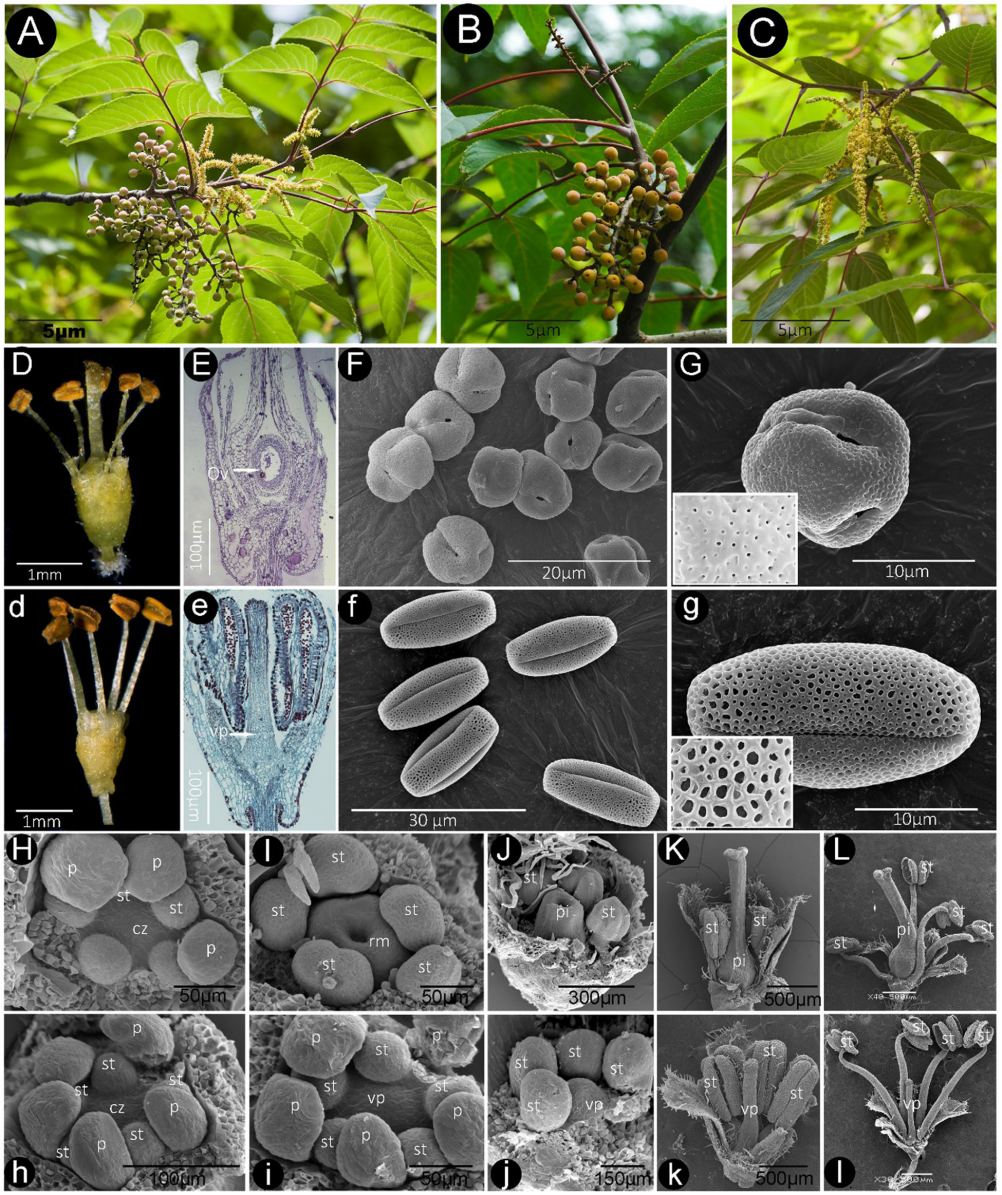
Floral morphology and development of *Tapiscia sinensis*^5,6^. **A** Hermaphroditic *T. sinensis* individual showing the simultaneous occurrence of flowers and fruit. **B** Hermaphroditic *T. sinensis* individual with fruits of the previous year and fruits of the current year developing on the same branch. **C** Male inflorescence on a male individual. **D**–**G** Hermaphroditic flowers; **D** one pistil shedding pollen and five anthers; **E** longitudinal section of a hermaphroditic flower showing the pistil and unilocular ovary; **F** spherical pollen; **G** the exine sculpture of pollen grains, showing details of the perforate tectum. **d**–**g** Male flower; **d** infertile pistil and five anthers; e longitudinal section of a male flower with no functional gynoecium; **f** olivary pollen; **g** the exine sculpture of pollen grains showing details of the reticulate tectum. **H**–**L**, **h**–**l** Scanning electron micrographs of flowers; hermaphrodite (**H**–**L**) and male (**h**–**l**) at various developmental stages; carpel primordium phase of hermaphroditic (**H**) and male (**h**) flowers. Gynoecial ridge stage of hermaphroditic (**I**, **J**) and male (**i**, **j**) flowers (note: we did not observe a gynoecial ridge stage of male pistil development; five petals were removed to make the gynoecium visible). Fully differentiated but immature hermaphroditic (**K**) and male (**k**) flowers. Mature hermaphroditic (**L**) and male (**l**) flowers. cz, Central zone; Ov, ovary; p, petal; pi, pistil; rm, ring meristem; st, stamen; vp, vestigial pistil

We compared the morphological differentiation of male versus hermaphroditic flowers (Fig. 3). *T*. *sinensis* flowers consist of five stamens with greenish filaments and yellow anthers 1–2 mm in length. In hermaphroditic flowers, the ovary is unilocular with 1 ovule; the style is longer than the stamens in bisexual flowers (Fig. 3d, e). In male flowers, the ovary is present but vestigial (Fig. 3d, e). Scanning electron microscope (SEM) images showed that there were no obvious differences between male flowers (MFs) and hermaphroditic flowers (HFs) before stage 5 (Fig. 3H, h). At stage 6, however, in hermaphroditic flowers, a ring meristem forms as a ridge around the central zone of the flower (Fig. 3I, J). At stage 7, the gynoecium grows as a hollow tube (Fig. 3J); from stages 11 to 14 (during flowering), the gynoecium becomes ready for fertilization, and when the stamens extend out from the petals, fertilization occurs (Fig. 3K, L). In contrast, at stage 6, in male flowers, the primordium of the gynoecium begins to form as a small bulge (Fig. 3i, j), unlike the apex of bisexual flowers (Fig. 3I). Male flowers produce a solid, nonfunctional, pistil-like structure (Fig. 3e, k). Bisexual flowers shed spherical pollen grains with a perforate tectum (Fig. 3F, G), while male flowers produced truncate pollen grains with a reticulate tectum (Fig. 3f, g).

### Selection associated with the sex-linked genomic regions of *T. sinensis*

**Fig. 4:**
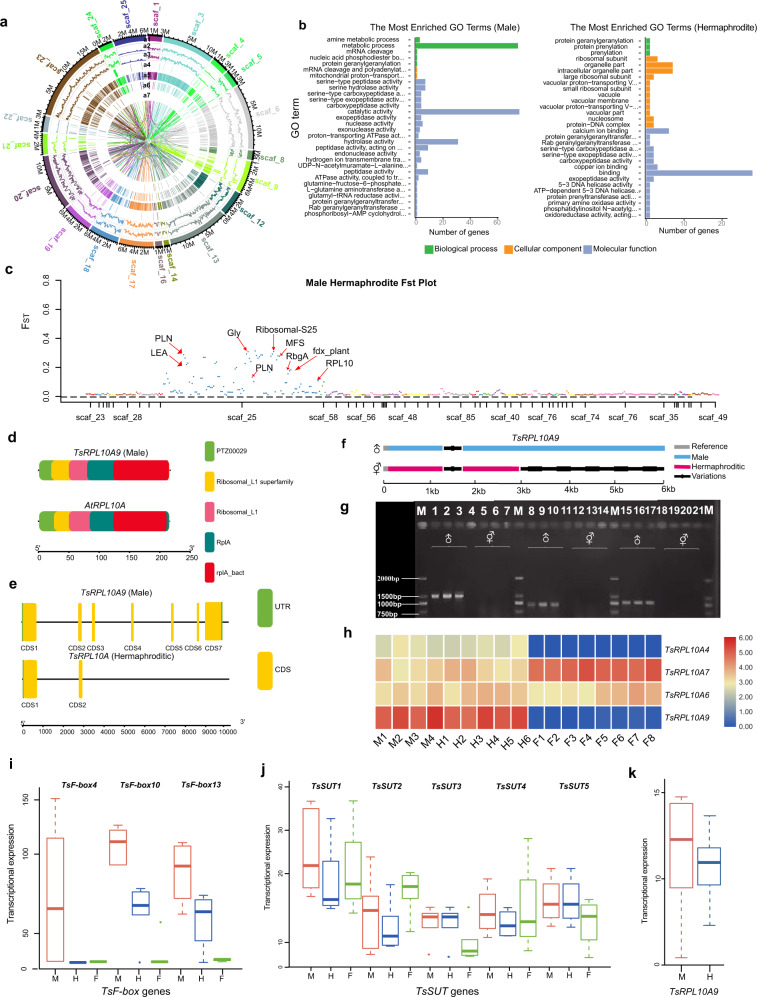
Candidate genes associated with sex-linked genomic regions of *T. sinensis*. **a** Alignment of genomic regions differentially enriched in male versus hermaphroditic groups of *T. sinensis*. Concentric rings, from outermost to innermost, include “a1”–Scaffolds, “a2”–SNPs, “a3”–Indels, “a4”-CNV duplications, “a5”–CNV deletions, “a6”–SV deletions, and “a7”–SV inventions. **b** The GO enrichments of genes existed only in males and hermaphrodites. **c** A Manhattan plot showing signatures of sequence divergence between male and hermaphroditic *T. sinensis*; histograms along each scaffold were based on a 20 kb window. The dots of different colors indicate regions of selective sweeps associated with sex linkage. Red arrows identify candidate genes with high and significant *F*_ST_ values between the genomes of pools of male and hermaphroditic *T. sinensis*. **d** Comparison of the *RPL10A* motif composition of *A. thaliana* and *T. sinensis*. **e** Gene structure of the *RPL10A* gene in male and hermaphroditic genotypes of *T. sinensis* compared with *A. thaliana*. **f** Molecular structure of *TsRPL10A-M* (male) and *TsRPL10A-H* (hermaphrodite) alleles verified by Sanger sequencing [the locations of primers (see Supplementary Table 22) are indicated by a light blue line (male) and light red line (hermaphroditic)]. The black line indicates variation (missing sequences) in hermaphroditic *T. sinensis*. **g** The PCR-based products of three male individuals and three hermaphroditic individuals using Sanger sequencing data. Three deletions (978 bp, 659 bp, and 773 bp) within *RPL10A* were found in hermaphroditic individuals (lanes 4 to 6, 11 to 13, and 19 to 21) compared with male individuals (lanes 1 to 3, 8 to 10, and 15 to 17). Lanes 4, 11, and 18 were controls (purified water was used as the template). **h** Tissue-specific expression of *RPL10A* genes identified in male flowers (M), hermaphroditic flowers (H), and fruits (F) of *T. sinensis* during the 8 months immediately following fertilization (for details, see Supplementary Table 23 and Supplementary Fig. 13). *RPL10A* genes and their expression values across different tissues are visualized as a heatmap. Genes associated with differences between the genomes of male and hermaphroditic plants are shown as bars (details on the sampled tissues are provided in Supplementary Table 23). **i** The mean expression of three *TsF-box* (*TsF-box4*, *TsF-box10*, and *TsF-box13*) genes identified in four male flowers (M), six hermaphroditic flowers (H), and eight fruits (F) of *T. sinensis* (for details on the sampled tissues, see Supplementary Table 24 and Supplementary Fig. 15). **j** The mean expression of each of five *TsSUT* (*TsSUT1*, *TsSUT2*, *TsSUT3*, and *TsSUT5*) genes in male flowers, hermaphroditic flowers, and fruits of *T. sinensis* (for details, see Supplementary Table 24 and Supplementary Fig. 14). **k** The mean expression of the *TsRPL10A9* gene in male flowers (M) versus hermaphroditic flowers (H) of *T. sinensis* (for details, see Supplementary Table 23 and Supplementary Fig. 13)

To identify sex-linked genomic regions of *T. sinensis*, we compared the genomes of 26 hermaphroditic individuals and 29 male individuals sampled from a variety of populations (Supplementary Table 18). Using *F*_ST_ values (cutoff set to 10E−5), we identified 303 candidate sex-linked genes (Fig. 4a, b and Supplementary Table 21). A high proportion (*N* = 79, 26.1%) of these candidates were located on scaffold 25 (Fig. 4c and Supplementary Table 21). The genes in males with the greatest *F*_ST_ were involved in amine metabolism, catalytic activity, and hydrolase activity. In hermaphroditic plants, the genes with the greatest *F*_ST_ were related to RNA binding and calcium ion binding. Other genes on scaffold 25 of hermaphroditic plants included phospholamban (*PLN*), late embryogenesis abundant (*LEA*), glyoxalase (*Gly*), major facilitator superfamily (*MFS*), ribosomal-S25 (*RPL*), 50S ribosome-binding GTPase (*RbgA*), ferredoxin (*Fdx*), and ribosomal protein l 10A (*RPL10A*) (Fig. 4c and Supplementary Table 21). We chose to analyze these loci further for insights into the genomic basis of androdioecy in *T. sinensis*. Among the 303 candidate sex-linked genes, we found that 64, 157, and 77 genes were expressed at significantly different levels in male flowers, hermaphroditic flowers, and fruits, respectively (Supplementary Fig. 8 and Supplementary Table 21). We further found that 34 of 303 genes (11.2%) had *F*_ST_ values >0.2, nine of which were highly expressed in male flowers, seventeen of which were highly expressed in hermaphroditic flowers, and eight of which were highly expressed in fruits (Supplementary Fig. 8 and Supplementary Table 21).

A total of 13 *TsRPL10A* genes were identified in the genome of *T. sinensis* using a search based on the conserved domain of *A. thaliana RPL10A*. The *TsRPL10A*s were unevenly distributed among 11 scaffolds, but only *TsRPL10A9* was located on scaffold 25 of *T. sinensis*. We analyzed the conserved domains and gene structure of the ribosomal_L1 superfamily *RPL10A* gene in male and hermaphroditic plants of *T. sinensis* and compared them to those in *A. thaliana* (Fig. 4d, e). The results showed that the *TsRPL10A9* gene of male *T. sinensis* was similar to the *A. thaliana RPL10A* gene; by contrast, several large deletions were present in the same locus of hermaphroditic plants of *T. sinensis* (Fig. 4d, e and Supplementary Figs. 9–12 and Supplementary Table 22). We verified the sequences of *TsRPL10A9* (male) and *TsRPL10A9* (hermaphroditic) by Sanger sequencing (for details of the six primer pairs used to span the gene, see Supplementary Table 22 and Supplementary Figs. 9–12). We performed a sequence alignment of *TsRPL10A9* (male) and *TsRPL10A9* (hermaphrodite) with the reference genome and annotated the resulting combination with gene structure analysis (Fig. 4d–g and Supplementary Figs. 9–12). The first 2660 bp of *TsRPL10A9* were identical between male and hermaphroditic plants, and all seven coding sequences (CDS1 to CDS7) of *TsRPL10A9* were present in male trees. In contrast, there were three deletions in exons of hermaphrodites that totaled 2410 bp [978 bp, 659 bp, and 773 bp located at CDS5 (5,423,343–5,423,399), CDS6 (5,424,898–5,424,977), and CDS7 (5,425,067–5,426,207), respectively]. These deletions in hermaphroditic plants were the major sources of sequence variation in *TsRPL10A* genes between male and hermaphroditic plants (Fig. 4f–h and Supplementary Figs. 9–12).

We examined the expression of *TsRPL10As* in *T. sinensis* tissues, including male and hermaphroditic flowers (Fig. 4h and Supplementary Tables 23, 24). Interestingly, most *TsRPL10A* genes were highly expressed in *T. sinensis* tissues, including fruits, male flowers, and hermaphroditic flowers (Fig. 4h and Supplementary Fig. 13), but two *RPL10A* genes (*TsRPL10A4* and especially *TsRPL10A9*) showed higher expression in male and hermaphroditic flowers than in other tissues (Fig. 4h). Moreover, *TsRPL10A9* was expressed at higher levels in male flowers than in hermaphroditic flowers (Fig. 4k).

*F-box* and sucrose transporter (*SUT*) family genes had high *F*_ST_ values, and members of these gene families were also on scaffold 25. For that reason, we investigated the transcript expression patterns of *F-box* and *SUT* family genes in *T. sinensis* tissues (Fig. 4i, j and Supplementary Fig. 14 and Supplementary Tables 23, 24). We found that three *F-box* genes (*TsF-box4*, *TsF-box10*, and *TsF-box13*) and two *TsSUT* genes (*TsSUT1* and *TsSUT4*) were differentially expressed in male versus hermaphroditic flowers of *T. sinensis* (Fig. 4j and Supplementary Fig. 14 and Supplementary Tables 23, 24). Nine *TsF-box* genes (*TsF-box*1, *TsF-box2, TsF-box5*, *TsF-box7*, *TsF-box9*, *TsF-box13*, *TsF-box14*, *TsF-box19*, and *TsF-box22*) contained a conserved self-incompatibility (*S-locus*) domain (Supplementary Fig. 15), although self-incompatibility has not been investigated in *T. sinensis*. We found that the expression of *TsRPL10A2*, *TsRPL10A7*, *TsRPL10A11*, *TsF-box14*, and *TsSUT4* was low in hermaphroditic individuals at stage 6 (Supplementary Figs. 13–15).

### Fruit quiescence in *T. sinensis*

**Fig. 5:**
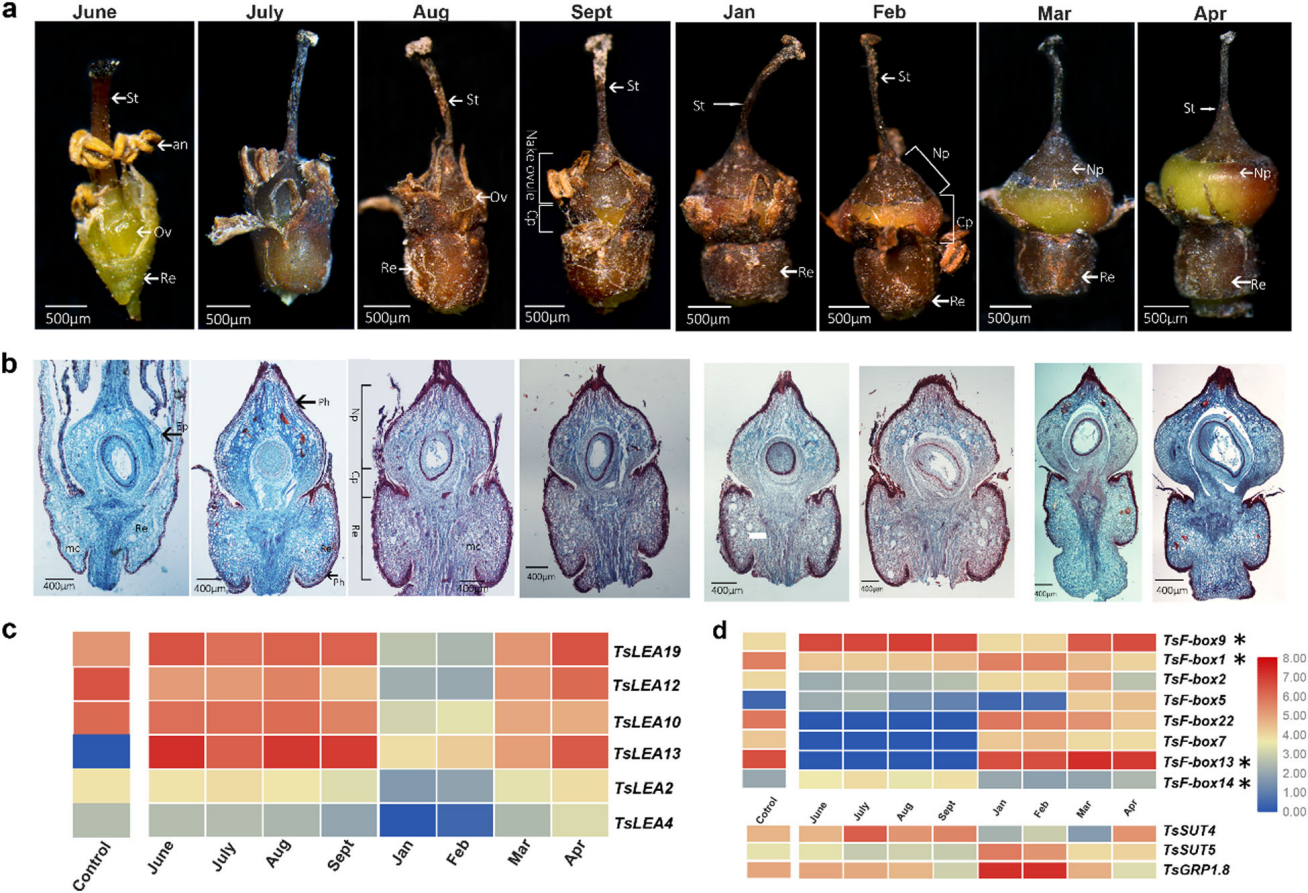
The morphology, anatomy, and gene expression levels of *T. sinensis* fruit during the 8 months immediately following fertilization. **a** The morphological characteristics of young fruits; actively growing fruits appear greenish in color, and quiescent fruits appear brown. **b** Structure of *T. sinensis* fruits over 8 months of development. **c** Tissue-specific expression of *LEA* gene family members in *T. sinensis* over 8 months (as in **a**, **b**) of fruit quiescence and development visualized as a heatmap. Transcript expression in flowers was used as a control. The fruit tissues were collected from January to August, and details are described in Supplementary Table 24. The genes shown were among those with high *F*_ST_ values between male and hermaphroditic plants, as shown in Supplementary Fig. 16. **d** Tissue-specific expression of *F-box* and *SUT* gene family members in *T. sinensis* over 8 months of fruit quiescence and development. an, anther; Cp, covered part of fruit; cz, central zone; ep, epidermis; mc, mucilaginous locule; Np, naked pericarp; Ov, ovary; Ph: phellem; Re: receptacle; st, stamen

The bisexual flowers of *T*. *sinensis* bloom in June, but *T. sinensis* takes 17 months to complete the cycle from floral primordium initiation (in March or April) to fruit maturation. After fertilization, the young fruit develops rapidly. The receptacle becomes fleshy and swollen, wrapping the ovary and forming a gourd-shaped structure. Young fruits become quiescent in August; from September to February, fruits are in deep quiescence (Fig. 5a), and their morphology and size remain unchanged until mid-April of the following year. In late March, flower development is initiated, and fruits reinitiate development; thus, flowers and fruits grow synchronously on hermaphroditic individuals (Fig. 3a). By the end of June, the current season’s fruits and previous year’s fruits are found on the same branch (Fig. 3b). Two months later (mid-August), the fruits of the preceding year are fully mature, and young fruits begin their quiescent period.

At the time of flowering, the ovary wall is composed of an inner and outer epidermis and 6–7 layers of parenchyma cells (Fig. 5b). In July, the parenchyma cells under the epidermis of the ovary wall develop a cork cambium that undergoes periclinal division to form phellem and phelloderm (Fig. 5b). In August, the phellogen cells in the ovary wall undergo periclinal division to form 3–6 layers of cork cells (outward) and one layer of phelloderm cells (inward) (Fig. 5b). The parts of the fruit protected by the remnant of the perianth and receptacle (“covered”) do not form periderm (Fig. 5b). Abundant mucilage locules form in the naked pericarp and, at the same time, in the whole receptacle (Fig. 5b).

The molecular mechanisms underlying the regulation of *T. sinensis* fruit quiescence and embryo development are likely to be complex and strongly temporally regulated. To understand the regulation of the development of *T. sinensis* fruit, we integrated genomic and transcriptomic analyses of genes in *T. sinensis* fruit at eight stages of quiescence and development (Fig. 5 and Supplementary Tables 21, 25, 26 and Supplementary Figs. 16, 17). Of the genes with high *F*_ST_ values between male and hermaphroditic plants, we focused on the *LEA* protein gene family, *TsF-box* genes, and *TsSUT* genes. Six *TsLEA* genes (*TsLEA2*, *TsLEA4*, *TsLEA10*, *TsLEA12*, *TsLEA13*, and *TsLEA19*), three *F-box* genes (*TsF-box5*, *TsF-box9*, and *TsF-box14*), and a *TsSUT4* gene were expressed at lower levels during fruit quiescence than at later times when fruits were actively developing (Fig. 5c, d). We found that the glycine-rich protein (*GRP*) *TsGRP1.8* gene was expressed at significantly higher levels during fruit quiescence compared with stages of active development (Fig. 5b, d and Supplementary Figs. 16, 17).

## Discussion

### Evolution of the androdioecious tree *T. sinensis*

The genome of the androdioecious tree *T. sinensis* was assembled and evaluated using 154 Gb of clean read data (343X). The final estimated genome size was 410 Mb, among the smallest reported for any tree. The genome was preliminarily annotated using RNA-seq data, from which we identified 22,251 protein-coding genes. Our study included a nearly complete representation and localization of genes, repeat elements, WGD, and RNAs; functional and metabolic annotations; and an analysis of the expression of genes with a putative role in sex determination and fruit development. A comparison of the genome of *T. sinensis* with the genomes of 17 angiosperm plants revealed differences in evolutionary expansion and contraction of gene families, in addition to unique gene families. These data represent an important resource for the study of evolutionary processes, gene evolution and function, molecular genetics and biochemistry in *Tapiscia* and the *Huertales*.

Currently, the genus *Tapiscia* comprises a single species, *T. sinensis*^1–5,24^, an endangered (Red List) species found in scattered, disjunct populations in subtropical and southwestern central China^25–27^. The *T. sinensis* habitats in the mountains of subtropical China were fragmented by past climatic changes^25^. Our results based on whole-genome resequencing showed that extant populations have low genetic diversity, are genetically distinct, and show little evidence of genetic exchange among populations (Fig. 2). The same conclusions were reached by Zhang et al.^25^ based on chloroplast haplotypes. It is possible that fragmentation of *T. sinensis* populations has resulted in populations with unique adaptations (Fig. 2). We suggest that populations of *T. sinensis* in the Tianmu Mountains, the Qinling Mountains, the Shennong Mountains, the Naling Mountains, and Yunnan Province be given the highest priority for conservation (Fig. 2).

### The transcriptomic and developmental bases of androdioecy

Androdioecy is a rare breeding system (occurring in <0.005% of angiosperms) in which populations consist of males and hermaphrodites^10^. The genetic and genomic bases of sex determination in plants are a critical area of study in biology and evolution. Although sex-linked systems are important in some horticultural plant species, such as red bayberry^29^, melons^30,31^, cucurbit^32^, kiwifruit^33^, and persimmons (*OGI*)^34^, the genomic and genetic bases of androdioecy have not been explored. We identified genomic regions and genes potentially associated with androdioecy by comparing male versus hermaphroditic genome pools. We identified a genomic scaffold (scaffold 25) that was unusually rich in candidate genes (Fig. 4 and Supplementary Tables 18–21). A 24-kb region of scaffold 25 was absent in hermaphroditic individuals. To confirm the function of this 24-kb region and determine its role in the specification of floral sexual development, a more complete, higher-quality genome assembly will be required^35–38^. We showed that the structure and sequence of *RPL10A* differed strongly between male and hermaphroditic trees, consistent with studies showing that *AtRPL10A* mutations caused female gametophyte lethality in Arabidopsis^39,40^, and that there were highly significant differences in the expression patterns of candidate *TsF-box* and *TsSUT* genes in male versus hermaphroditic flowers and fruit (Fig. 4).

In previous studies, our results showed that bisexual flowers are the ancestral condition in *T. sinensis*^4–6^. Loss of female function may be the consequence of a large-scale insertion into *TsRPL10A* that caused the development of a sterile pistil in the hermaphroditic flower primordium (Figs. 3 and 4)^39^. *T. sinensis*, as an androdioecious tree, may maintain characteristics associated with self-incompatibility^41^. We identified self-incompatibility-related *F-box* genes in the *T. sinensis* genome, including *TsF-box14*, *TsF-box19*, and *TsF-box22*, which contained a conserved self-incompatibility (*S-locus*) domain^42^ whose function might be confirmed by transformation into a model such as *A. thaliana* or *Nicotiana tabacum*^43,44^.

*Tapiscia sinensis*, although mostly subtropical in its distribution, also occupies habitats where temperatures can fall as low as –20 °C. Cold hardiness evolves through long-term adaptation to low-temperature environments^45–49^. *T*. *sinensis* flowers are fertilized in early June, the young fruits become quiescent in September, and this status is maintained until the following April. Quiescent young fruits have several characteristics that appear to be adaptations to low temperatures, including phellem formation on the ovary surface, an ovary enclosed in a receptacle, zygote quiescence, and protein, carbohydrate and lipid accumulation in the “overwintering complex”^1,50^. These developmental traits, along with androdioecy, may be adaptations to long-term climatic oscillations^1–5,24^. Among the genes that differed most between male and bisexual flowers, several had potential roles in cellular protection under cold stress or increasing storage proteins^51^. The expression levels of *F-box* genes (*TsF-box1, TsF-box22*, and *TsF-box13*) and *SUT* genes (e.g., *TsSUT5*) were higher at times, consistent with a role in fruit development and quiescence^52–54^ (Fig. 5 and Supplementary Figs. 14, 15). We further showed that the *TsGRP1.8* gene was highly expressed during fruit quiescence (Fig. 5d). The *GRP1.8* gene is part of a repair system during the stretching phase of protoxylem development^53^ and insolubilized within the cell wall later in development^54^ (Fig. 5 and Supplementary Fig. 17). It is possible that *TsGRP1.8* plays a role in increasing the cell wall thickness or fruit stability to survive cold temperatures during quiescence (Fig. 5b, d)^55^.

The pollen of bisexual flowers of *T. sinensis* is round and unlike that of male flowers, a phenomenon also observed in *Tetracera oblongata*, which is functionally dioecious^56^. This may indicate that pollen of hermaphroditic plants of *T. sinensis* is nonfunctional, a possibility suggested by Charlesworth (1984) as likely based on theoretical considerations^57^. Charlesworth^58^ also considered the 1:1 ratio of male to fruitful plants that we observed to be evidence that hermaphroditic plants were male sterile. We did not observe whether hermaphrodite pollen was fertile but did observe that hermaphrodite pollen can be shed onto adjacent stigmas, an event that would lead to inbreeding unless there is a self-incompatibility system, which we did see evidence of in nine *F-box* genes (Supplementary Fig. 15). Charlesworth agreed with Sobrevila and Arroyo (1982), who speculated that in *Matayaba tovarensis*, dioecy evolved from self-incompatibility in hermaphrodites^59^. Charlesworth (1984) further stated that “when pollen is actually produced in females, but is nonfunctional, a role for inbreeding avoidance seems particularly clear”, as sterile pollen can still attract pollinators. We did not record whether *T. sinensis* produces nectar as a reward, but *TsSUT3* was expressed in flowers but not fruit, so it could be related to nectar production (Supplementary Fig. 14 and Fig. 4i). We also observed that the expression of some *SUT* genes was consistent with a role in fruit development, which of course only happens in hermaphroditic plants. It makes sense that sucrose transporters would be needed for fruit development.

In conclusion, the genome assembly of *T. sinensis* provides a reference for future genetic, genomic and evolutionary studies in *Tapiscia* and its relatives. *T. sinensis* was fragmented into five lineages based on whole-genome resequencing data from 10 populations and 55 individuals. A 24-kb region located on scaffold 25 was absent in the hermaphroditic individuals. It was rich in candidate sex-linked genes, e.g., *TsRPL10A9*^52^. We further identified eight *F-box* genes, six *LEA* genes, four *SUT* genes, and one *GRP* gene that are involved in sugar metabolism in fruit, overwintering fruit development, and flower development^42–44,53–62^, potentially indicating that these genes play a role in regulating flower development and fruit quiescence during winter in *T. sinensis*. These genes may be of use for *T. sinensis* breeding or for further identification of genomic regions affecting sex determination. Finally, the identification of sex-linked genes in *T. sinensis* will inform studies on the evolution of androdioecy, sex-linked gene functional analysis, plant sexual development, and fruit development.

## Methods

### Tree samples

In 2016, we collected leaf samples from a single individual of the androdioecious tree *Tapiscia sinensis* (wild male individual) growing in the Qinling Mountains, Shaanxi Province, China (E108°35’05.60”, N33°31’22.70”). High-quality genomic DNA was extracted from fresh leaf tissues using the Qiagen DNeasy Plant Mini Kit (Qiagen, Dusseldorf, Germany). In 2016, shoots were collected from 55 trees representing 10 wild populations of *Tapiscia sinensis* in seven Chinese provinces (Supplementary Table 18). These 10 populations represented most of the geographic distribution of this endangered, woody tree species (Fig. 2).

### Transcriptome sample preparation and sequencing

Five tissues (fruits, leaves, stems, roots, and male flowers) were collected from a single male tree of *T. sinensis* (located in the Qinling Mountains, Shaanxi Province, China). We also collected hermaphroditic flowers from a hermaphroditic tree growing in the Qinling Mountains (E 108°35’, N 33°31’). RNA was extracted from these materials with TRI reagent. The RNA-seq data of six tissues were used to make libraries using the TruSeq® RNA Sample Preparation protocol for the Illumina HiSeq 4000 platform. Transcriptomes of 18 independent samples of tissues (four male flowers, six hermaphroditic flowers, and eight fruits) were evaluated for levels of expression using genes identified in *T. sinensis* (Supplementary Table 24).

### Library preparation

For Illumina sequencing (HiSeq 4000 platform), we used a paired-end library with an insert size of 350 bp. Paired-end 10X Genomics genome libraries with insert sizes of 50–100 kb were constructed using the Illumina NovaSeq platform. Long reads of 20 kb DNA inserts were constructed for PacBio single-molecule real-time sequencing to assist in genome assembly.

### Genome de novo assembly

Before sequence assembly, “daligner” was executed by the main script of the FALCON assembler to correct errors in the PacBio long reads and to generate consensus sequences^63^. After error correction, the consensus sequences achieved accuracies up to 99.999%. Then, FALCON identified the overlaps between all pairs of the preassembled error-corrected reads. The read overlaps were used to construct a directed string graph following Myers’ algorithm^64^. Contigs were constructed by finding the paths from the string graph (falcon_sense_option = --output_multi --min_idt 0.70 --min_cov 3 --max_n_read 200 --n_core 10; overlap_filtering_setting = --max_diff 100 --max_cov 100 --min_cov 3 --n_core 24)^64^. Error correction of the preceding assembly was performed using the PacBio “arrow” consensus–calling algorithm^65^. Illumina reads were error-corrected with Pilon^66^. FragScaff software was used to extend 10X Genomics scaffolds as follows: Linked reads generated using the 10X Genomics library were aligned to the consensus sequence of the PacBio assembly to obtain superscaffolds using BOWTIE v2.2^67^. As the length of the consensus sequence increases, fewer linked reads supporting connections are required. The consensus sequence with linked-read support was used for the subsequent assembly. The FragScaff parameters were “-fs1 ‘-m 3000 -q 30 -E 30000 -o 60000’ -fs2 ‘-C 5’ -fs3 ‘-j 1 -u 3’”^68^. We also evaluated the completeness and accuracy of the genome assembly using Bench Marking Universal Single-copy Orthologs (BUSCO) version 4.0.5^69^.

### Repeat annotation

We identified the repeat sequences in the *T. sinensis* genome using LTR_FINDER^70^, PILER-DF^71^, RepeatScout^72^, and MITE-Hunter^73^. The Repbase database^74^ was combined to construct a nonredundant repeat sequence library of the *T. sinensis* genome. Tandem Repeats Finder was used to identify tandem repeat sequences in the *T. sinensis* genome^75^. MISA software was used to identify the simple sequence repeats (SSRs), with thresholds of ten repeat units for mononucleotide SSRs and five repeat units for di-, tri-, tetra-, penta-, and hexanucleotide SSRs.

### Gene prediction and annotation

To predict genes in the *T. sinensis* genome, we used two methods: homology-based prediction with BLAST (E-value ≤ 1e^−5^)^76^ and GeneWise (version 2.4.1)^77^ and ab initio prediction with Augustus^78^ and GlimmerHMM^79^. We predicted the gene structure of the corresponding genomic regions using BLAST hits through GeneWise (version 2.4.1)^77^. Protein sequences were downloaded from the NCBI to perform homology predictions. RNA-seq data derived from six tissues (fruit, leaf, stem, root, male flower, and hermaphroditic flower) were assembled using Trinity. We generated gene models using Evidence Modeler (EVM)^80^. UTR and alternative splicing variation were predicted from gene models using PASA2 software^81^.

We performed functional annotation using BLASTP with an E-value cutoff of 1e^−5^. Protein-coding genes were identified with searches against SwissProt and TrEMBL^82^. We annotated conserved protein domains using the InterPro (version 5.16)^83^ and Pfam (v 3.0) databases^84^. We predicted the biological pathways in the KEGG database^85^ using BLAST with an E-value cut-off of 1e^−05^.

### Comparative analysis of *T. sinensis* genome evolution

We performed global gene family classification of 17 plant genomes, including those of *M. notabilis*, *Z. jujuba*, *P. persica*, *O. sativa*, *A. thaliana*, *J. regia*, *J. curcas*, *G. max*, *G. raimondii*, *T. cacao*, *F. schinensis*, *P. trichocarpa*, *V. vinifera*, *C. papaya*, *S. lycopersicum*, *O. europaea*, and *C. sinensis*, based on whole protein-coding gene repertoires. We performed gene pair searches within these 17 plant genomes using BLASTP. We clustered gene families from these plant species using OrthoMCL^86^ with the parameter “-inflation 1.5”.

### Phylogenetic tree reconstruction

We first generated a multiple sequence alignment with default parameters through MUSCLE^84^. We used ProtTest^87^ to select the best substitution models, and the JTT + I + G + F model was selected as the best-fitting model. Then, we constructed a maximum likelihood (ML) phylogenetic tree based on 509 families’ genes using RAxML^88^.

### Species divergence time estimation

We estimated the divergence times for 18 species using MCMCTree in PAML^89^ with the “correlated molecular clock” and “JC69” models. We applied a total of five fossil calibration points for the estimation of divergence times: *O. sativa* and *S. lycopersicum* divergence time (115–308 million years ago), *V. vinifera* and *G. max* divergence time (107–135 million years ago), *V. vinifera* and *A. thaliana* divergence time (107–135 million years ago), *J. regia* and *P. trichocarpa* divergence time (101–131 million years ago), and *P. trichocarpa* and *A. thaliana* divergence time (98–117 million years ago) (http://www.timetree.org/) (Fig. 1).

### Gene family expansion and contraction

CAFÉ 2.2^90^ (Computational Analysis of gene Family Evolution) software was used to assess the expansion and contraction of orthologous gene families among the 18 plant genomes based on a phylogenetic tree.

### Whole-genome duplication analysis

We identified syntenic blocks in the *T. sinensis* genome using MCScanX with a BLASTP E-value < 1e^−7^. We calculated the 4DTv distance for each gene pair in a syntenic block to identify putative whole-genome duplication events in *T. sinensis*. The synonymous substitution rate (Ks) was calculated using the YN model in KaKs_Calculator v2.0^91^.

### Transcriptome library and gene expression analysis

Transcriptomes (*N* = 18) were mapped to the *T. sinensis* genome using TopHat^92^. The expression of each gene was normalized by read counts (RPKM). We used DESeq to identify differentially expressed genes (Supplementary Table 26). We generated the expression profiles of all differentially expressed genes using gCLUTO (http://glaros.dtc.umn.edu/gkhome/cluto/gcluto/overview).

### Alignment and variation calling from whole-genome resequencing data of 55 accessions

We mapped the short reads to the *T. sinensis* genome using BWA with the following parameters: bwa mem -t 4 -M -R^93^. SAM/BAM files were evaluated to remove PCR duplicates using the Picard package with default parameters (http://broadinstitute.github.io/picard). We computed the coverage and depth of sequence alignments using the DepthOfCoverage program in the Genome Analysis Toolkit^94^ (GATK) and BEDtools^95^, respectively (Supplementary Table 19). We called SNPs following GATK best practices. Low-quality alignments with a mapping quality <20 were filtered using SAMtools^96^.

### Population genetic analysis

We constructed a neighbor-joining (NJ) tree of 55 *T. sinensis* accessions using TreeBeST^97^ and EvolView^98^. We performed PCA using GCTA software with default settings^99^. The top four eigenvectors of samples were plotted using the ggplot2 R package^100^. We also investigated population structure using ADMIXTURE^101^, wherein the maximum iteration time was set to 10,000, and the number of population groups (*K*) was varied from 1 to 10. We identified sex-linked genomic regions by comparing 26 hermaphroditic individuals and 29 male individuals for regions showing reduction of diversity (ROD = 1 − π_male_/π_hermaphroditic_). *F*_ST_ parameters were calculated in windows of 10 kb along the entire *T. sinensis* genome (Supplementary Table 18).

### Floral morphology and development

Living tissues were collected from cultivated trees growing at Northwest University, Shanxi Province (N34°14'58.61”, E 108°55'39.72”). Samples were collected in June, July, August, September, January, February, March, and April. We observed and photographed the morphological features of *T. sinensis* using a stereomicroscope (OLYMPUS SZX12). We prepared paraffin sections using routine methods^102^. Briefly, tissues of *T. sinensis* were dehydrated in an ethanol series (5 min 85% ETOH, 95% ETOH, 100% ETOH, and 100% ETOH), infiltrated with xylene (20 min 1/2 ETOH + ½ xylene and 15 min wash in 100% xylene two times)^4^, and embedded in paraffin for sectioning. The prepared slides were critically observed and photographed using a Nikon DS-Fil digital camera^5^.

### Gene families

Query sequences of *RPL10A*, *SUT*, *LEA*, and *F-box* genes of *A. thaliana* were downloaded from TAIR (https://www.arabidopsis.org/browse/genefamily/index.jsp). Gene family size for *RPL10A*, *SUT*, *LEA*, and *F-box* gene families was determined using blastp with the default parameter^70^. We characterized the structure of *RPL10A*, *SUT*, *LEA*, and *F-box* genes using the online software GSDS2.0^103^. The protein domains of these four gene families’ members were predicted in NCBI software. The R package pheatmap was used to detect the expression pattern of these four gene families (R package)^104^.

### PCR verification of *RPL10A* gene variations

Total genomic DNA of *T. sinensis* was extracted using the CTAB method^4,5^. We designed six *TsRPL10A* primer pairs to verify the genetic variation between male and hermaphroditic individuals (Supplementary Table 22). The PCR protocols were as previously reported^3–5^, using the previously mentioned primers (Supplementary Table 22), and the PCR products were verified by agarose gel electrophoresis.

## Supplementary information

Supplementaty Figures1–17

Supplementary Tables1–26

## Data Availability

The raw reads of the genome and the resequencing data for 55 *Tapiscia sinensis* individuals have been deposited as a BioProject under accession PRJNA587558. The raw transcriptome data for flowers and fruits of *T. sinensis* have been deposited as a BioProject under accession PRJNA284864.
