# Supplementary material for: The de novo genome assembly of Tapiscia sinensis and the transcriptomic and developmental bases of androdioecy
Source: Hortic Res. 2020 Dec 1;7:191. doi: 10.1038/s41438-020-00414-w (PMC7705024; doi:10.1038/s41438-020-00414-w)
Supplement: Supplementary file 1 — Supplementaty Figures1–17 [file 41438_2020_414_MOESM1_ESM.docx]

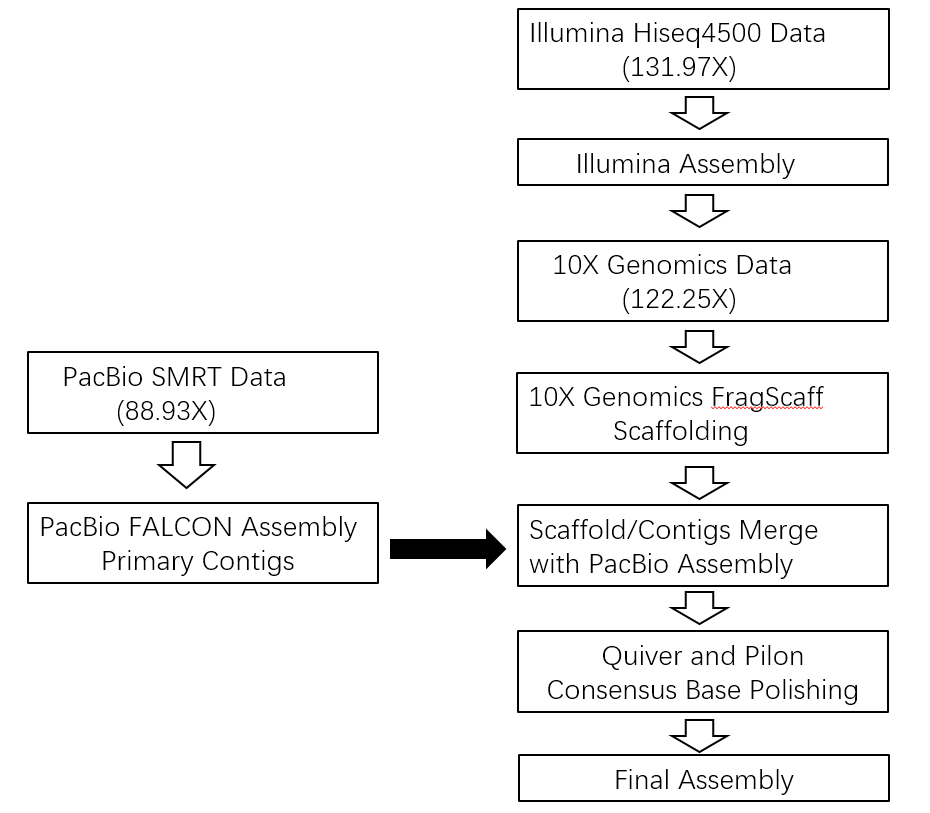


**Supplementary Fig. 1.** Genome assembly flowchart demonstrating assembly merge and data integration.


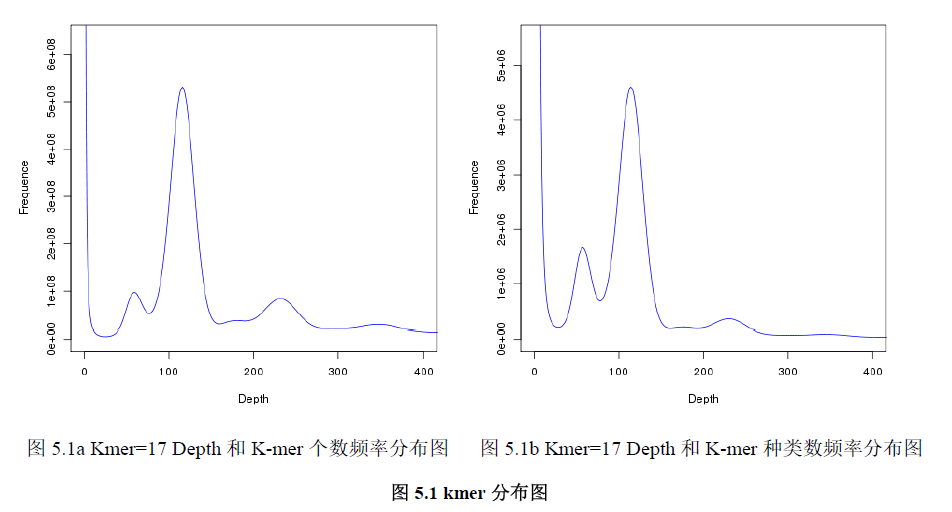


Supplementary Fig. 2. The distribution of 17-mer depth (L) and K-mer depth (R) of the Illumina PE reads. The analyzed reads from male libraries with insert sizes of 500 bp and 450bp after filtering raw reads and sequence error correction. According to *K-mer* frequency information, the peak depth was 115, and the genome size of *T. sinensis* was estimated as 428 Mb.


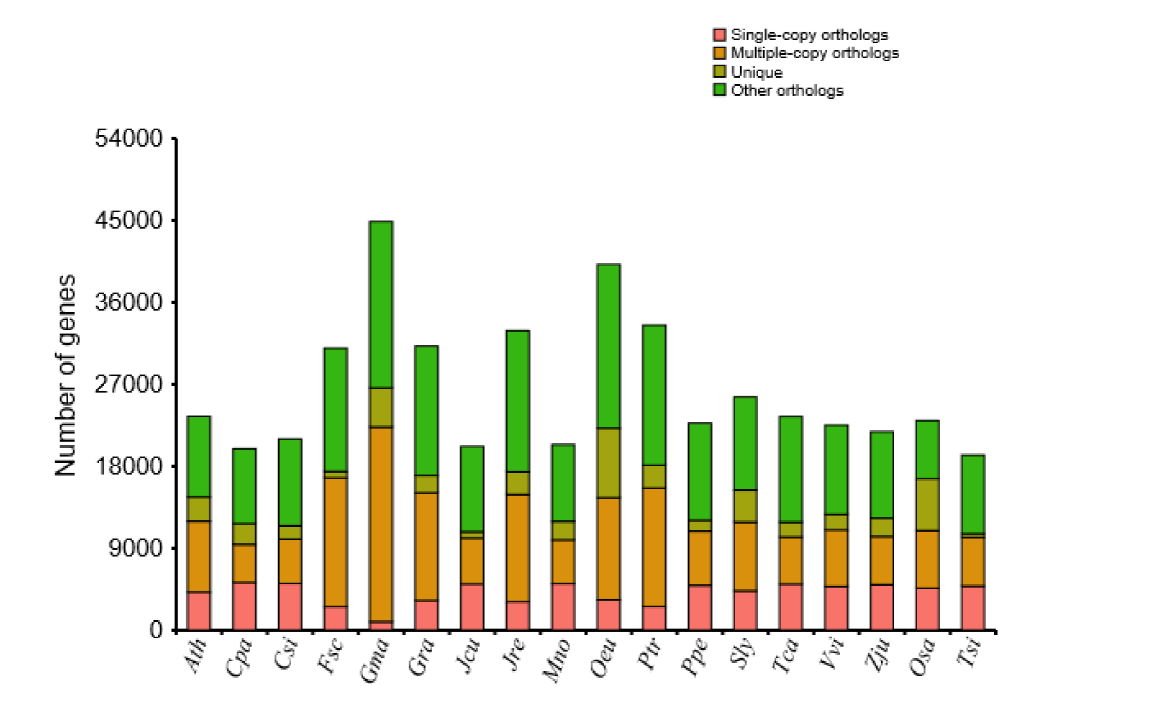


**Supplementary Fig. 3.** Comparisons of ortholog protein families among eighteen plant species. *A. thaliana*, *C. papaya*, *C. sinensis*, *F. schinensis*, *G. max*, *G. raimondii*, *J. curcas*, *J. regia, M. notabilis*, *O. europaean*, *P. trichocarpa*, *P. persica*, *S. lycopersicum*, *T. cacao*, *V. vinifera*, *Z. jujube*, *O. sativa*, *T. sinensis* were used for analysis. Species names are shown on the x-axis and the numbers of orthologous or paralogous sequences in the genome on the y-axis.


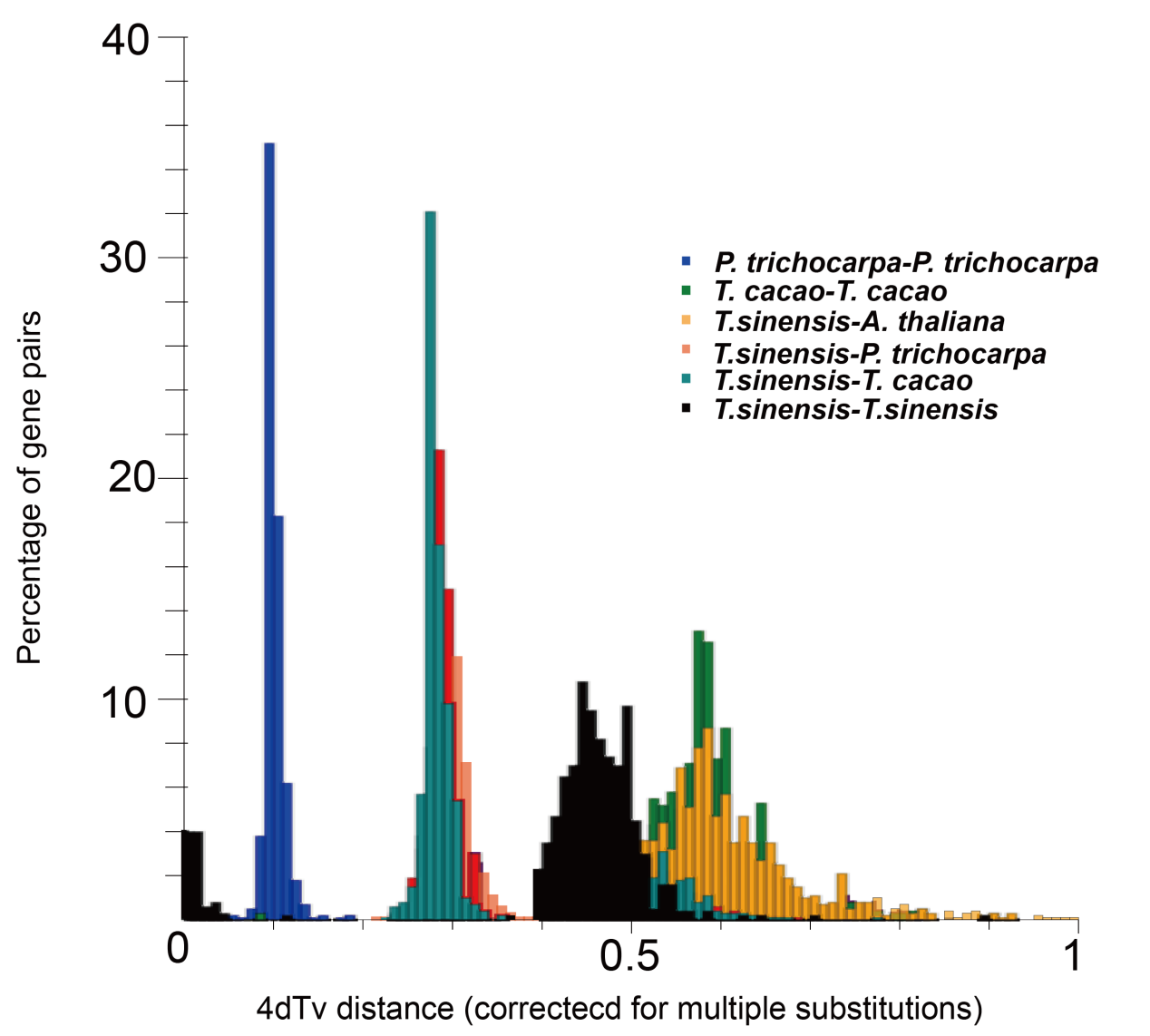


**Supplementary Fig. 4**. Distribution of 4dTv distances between duplicated genes of syntenic regions in *A. thaliana*, *P. trichocarpa*, *T. cacao*, and *T. sinensis* are shown by coloured plots as indicated.


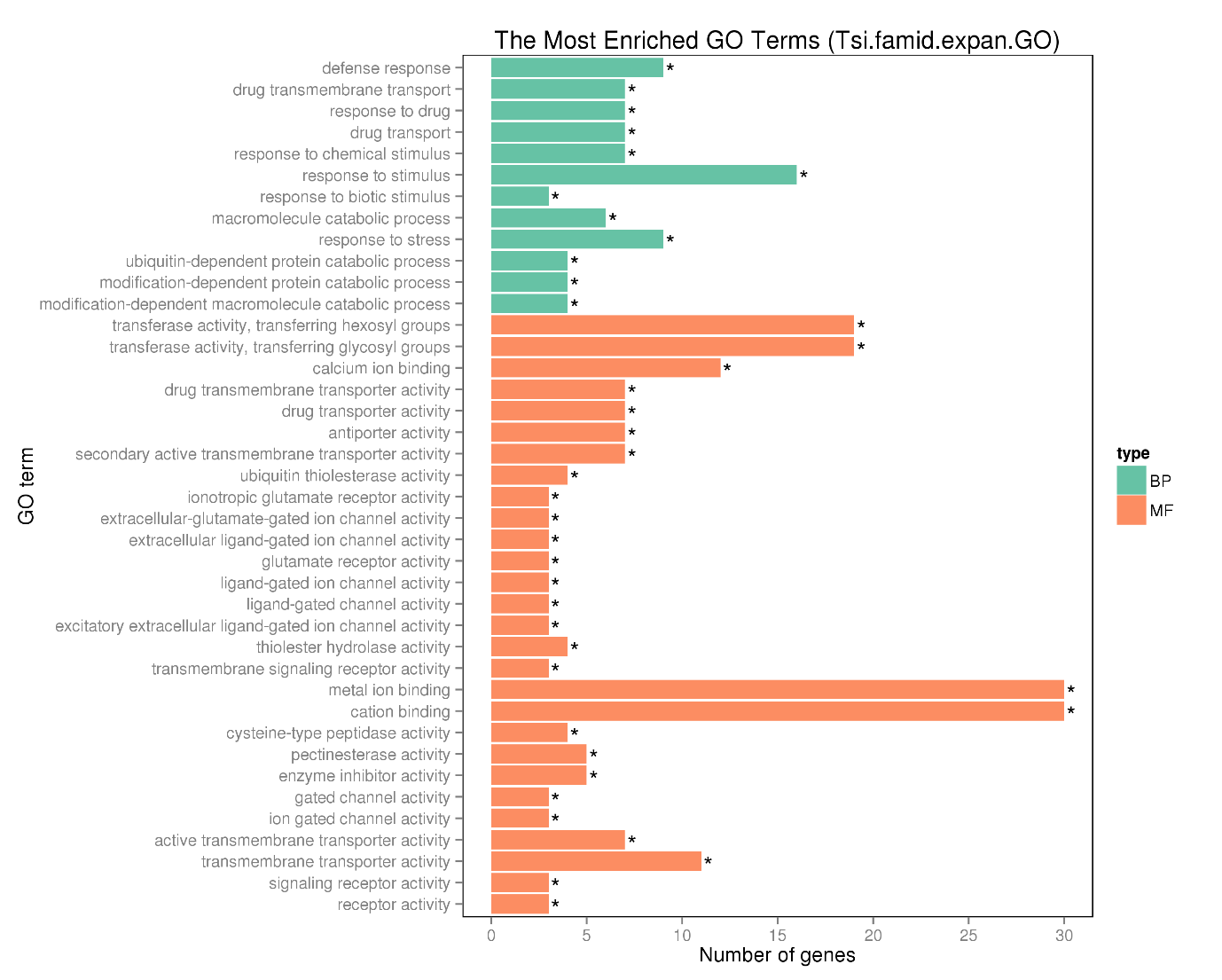


**Supplementary** Fig. 5. GO (Gene Ontology () annotations for gene families expanded during the evolution of *T. sinensis*.BP indicates that biologiacl process; MF indicates that molecular function.


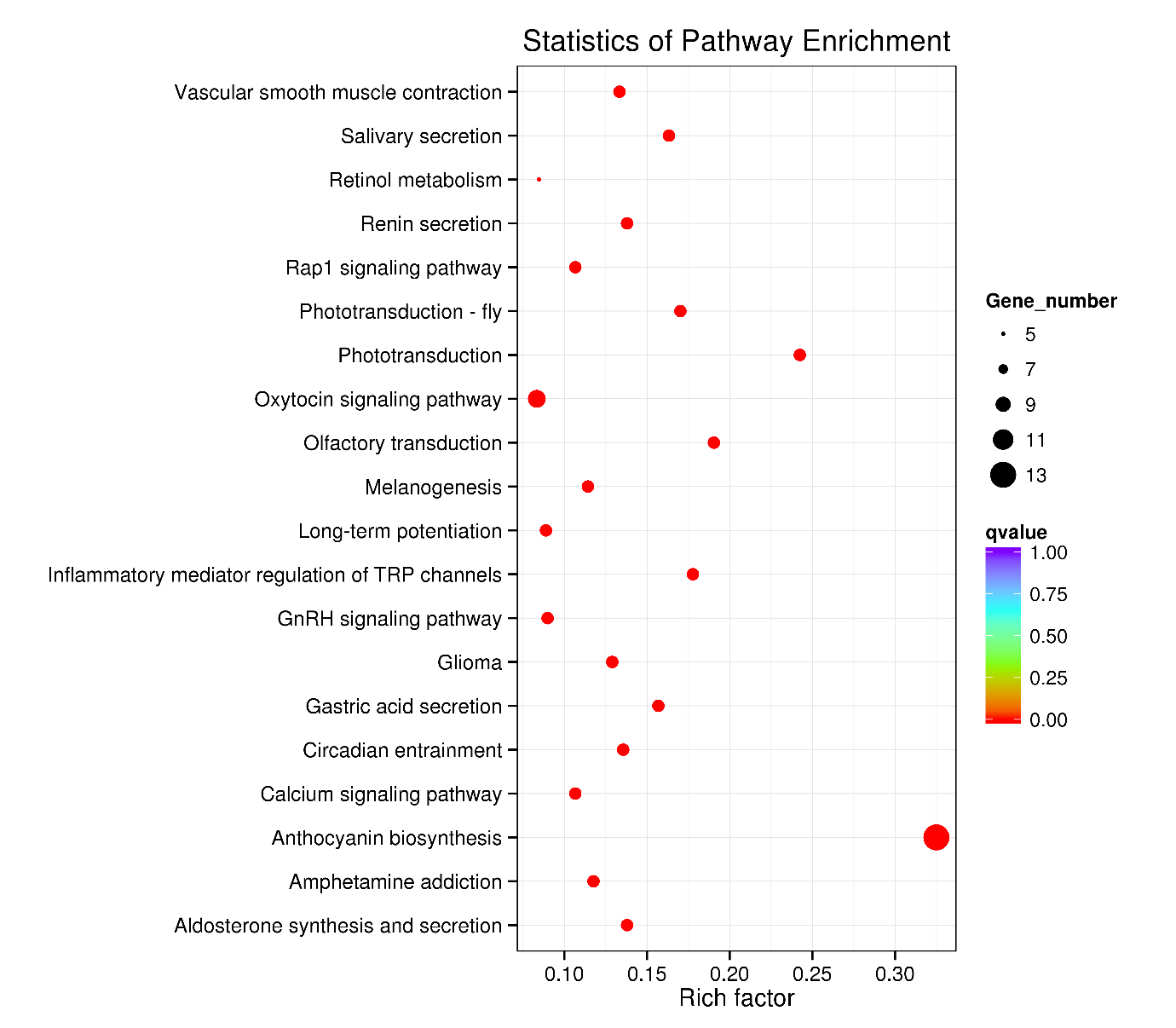


**Supplementary** Fig. 6. KEGG (kyoto encyclopedia of genes and genomes) annotations of genes expanded in *T. sinensis* relative to 18 other plant lineages, see Supplementary Fig. 4 for more details.


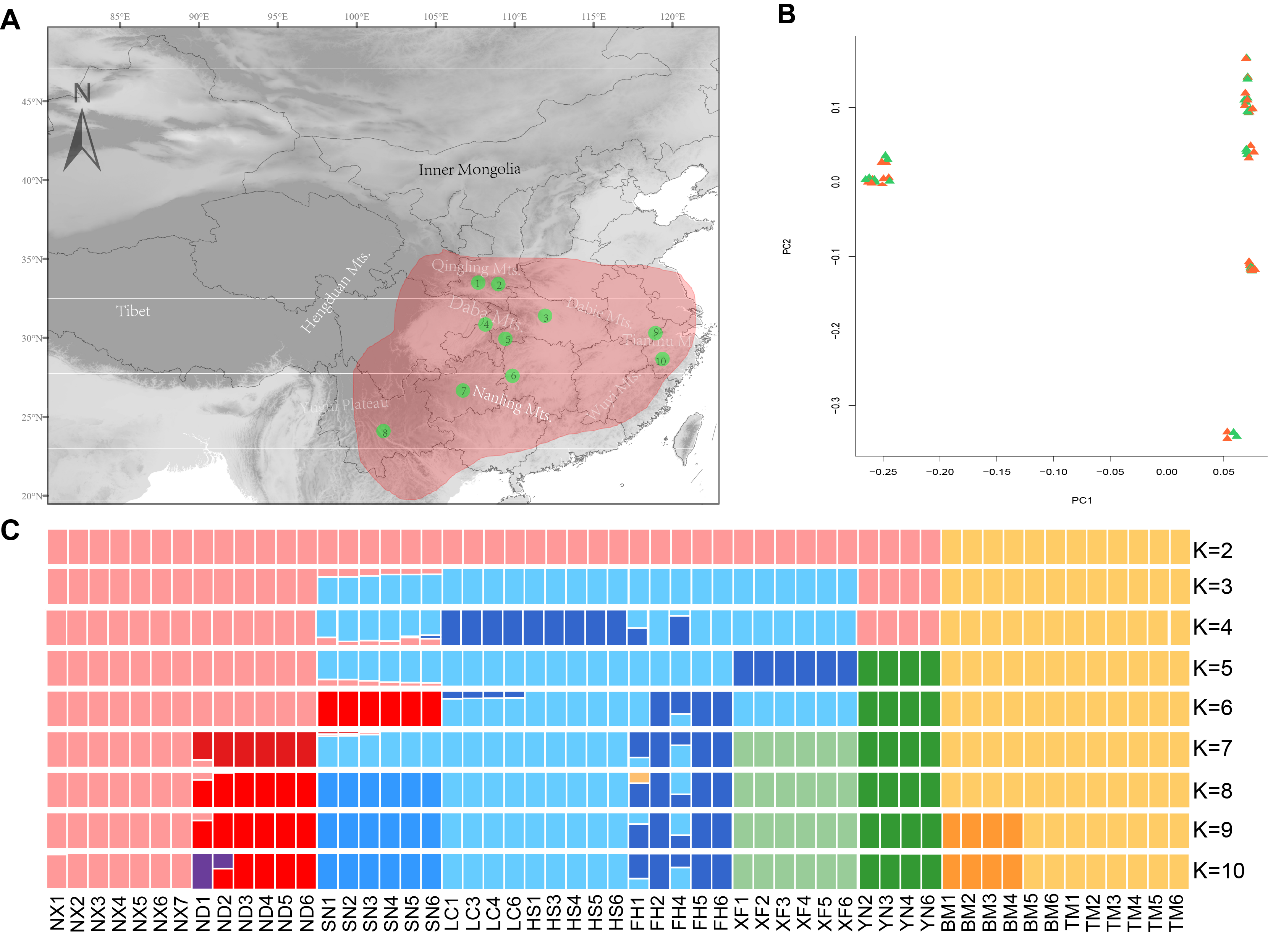


**Supplementary Fig. 7.** Whole genome resequencing to determine the genetic structure of wild *Tapiscia sinensis* populations. **a.** Locations of ten sampled populations. The number from 1 to 10 indicate ten populations in this study, for details, see supplementary Table 18. **b.** PCA analysis of 55 *T. sinensis* accessions using 11,431,841 SNPs based on whole genome resequencing. Red triangles indicate male trees and green triangles indicate the hermaphroditic trees. **c.** Results of STRUCTURE analyses of 55 individuals of *T. sinensis* from 10 sampled locations based on 11,431,841 SNPs, at K=2 to 10.


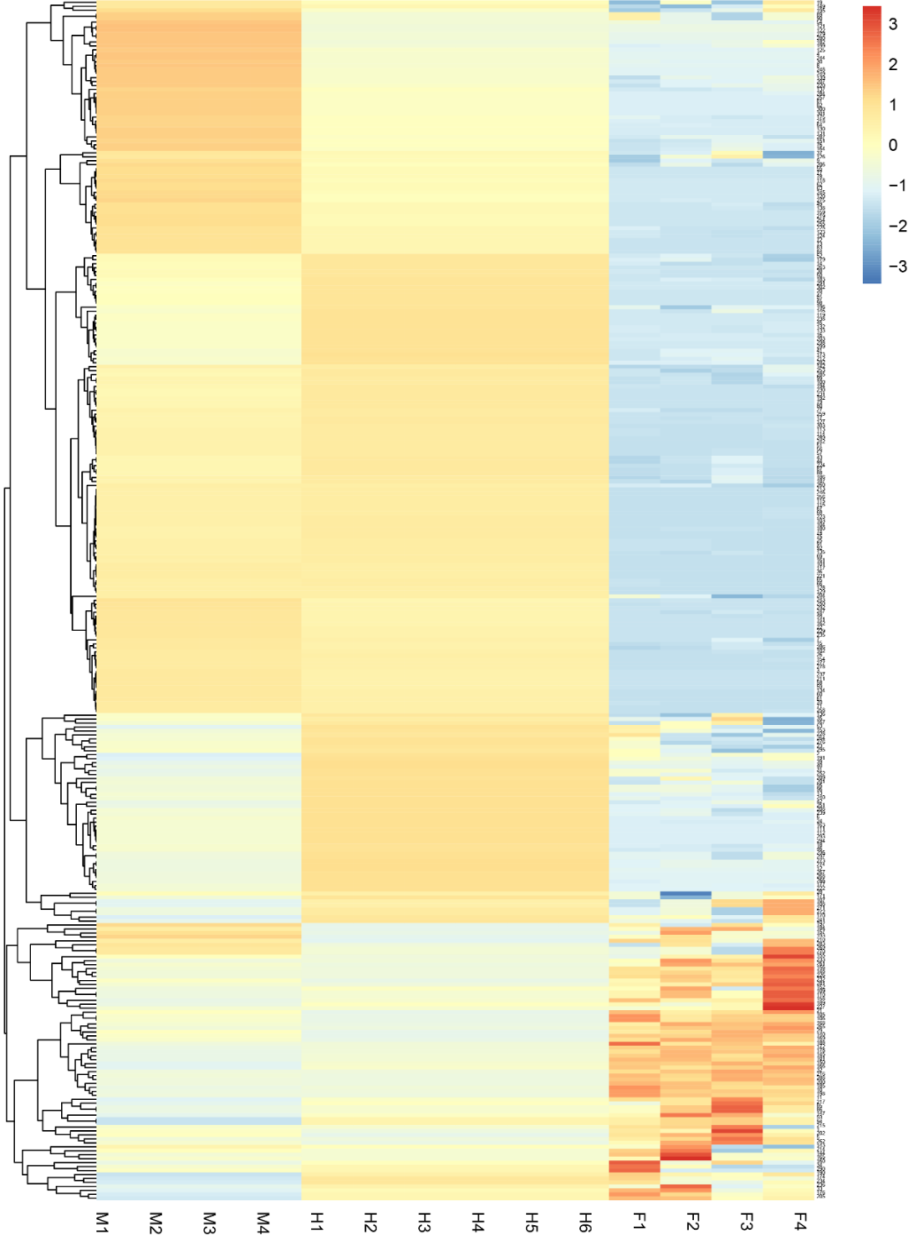


**Supplementary Fig. 8.** Expression in male flowers (M1 – M4), hermaphroditic flowers (H1 – H6) and fruit (F1 – F4) of 303 candidate sex-linked genes in *T. sinensis*. Details for the 303 genes are in Supplementary Table 21; details for transcriptome sequencing (RNA-Seq) and tissue samples are in Supplementary Table 24. Within 303 candidate sex determination genes, a total of 64 (red), 157 (blue), and 77 (green) genes showed significantly different expression in male flowers, hermaphroditic flowers, or fruits (details see Supplementary Table 21) Red indicates that these genes highly expressed in Male flower, blue indicates that these genes highly expressed in Hera flower, green indicates that these genes highly expressed in fruit.


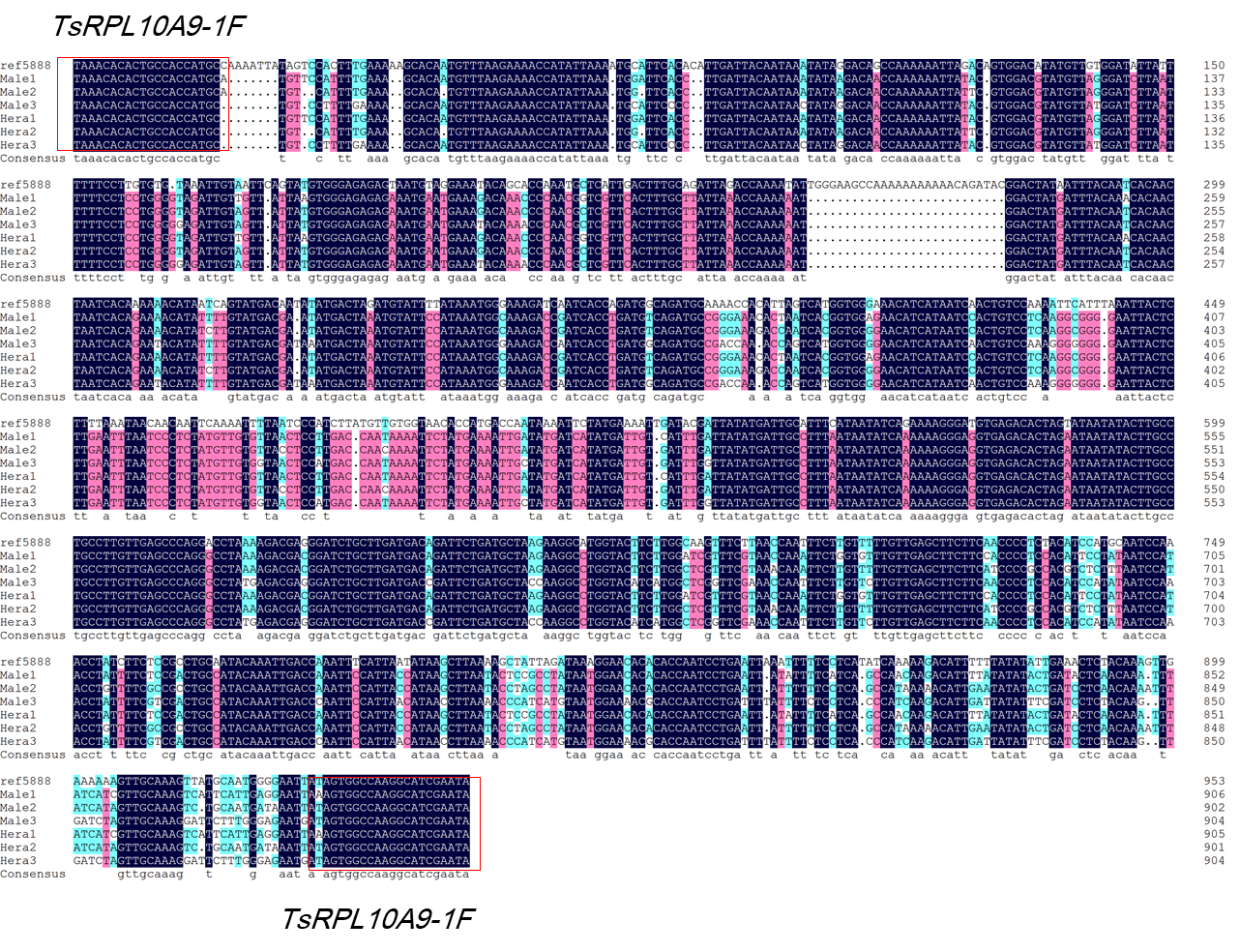


Supplementary Fig. 9. The first PCR Sequence alignment of male and hermaphroditic (Hera) trees at *RPL10A* aligned with the reference genome.Primer information details see in Supplementary Table 22. Ref: reference, consensus indicates that the same sequence. The Male 1, 2, 3 and Hera 1, 2, 3 were biological replicates indicates that leaf samples from different individual of the *Tapiscia sinensis* trees growing in the Qinling Mountains, Shaanxi province, China (E108°35’, N33°31’). Light blue indicates there were variation, cyan indicated few locus can not align with the others, black indicates that there were high similarity. Red rectangle indicates the primer location and sequence.


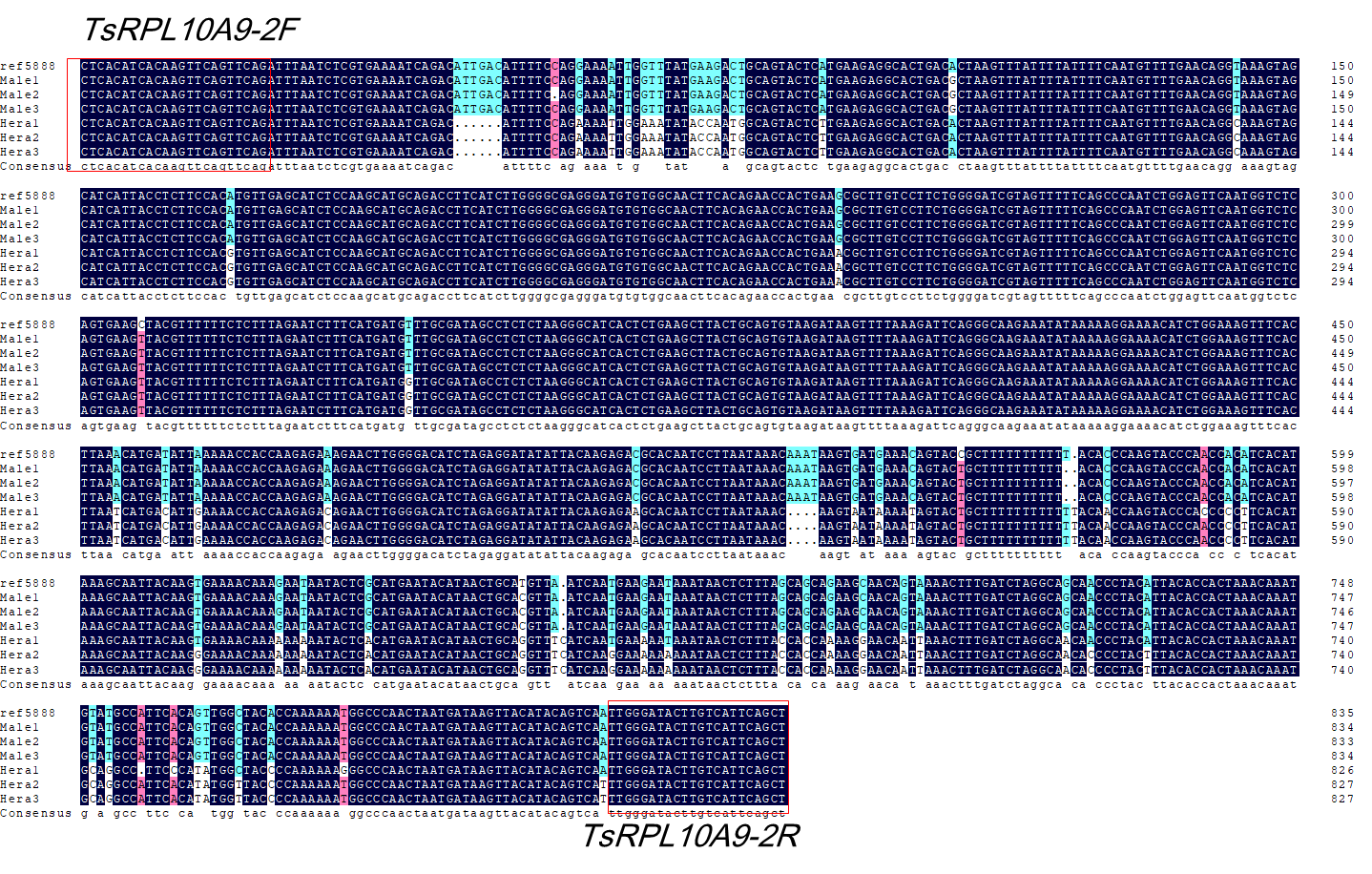


Supplementary Fig. 10. The second PCR Sequence alignment of male and hermaphroditic (Hera) trees at RPL10A aligned with the reference genome.Primer information details see in Supplementary Table 22. Ref: reference, consensus indicates that the same sequence. The Male 1, 2, 3 and Hera 1, 2, 3 were biological replicates indicates that leaf samples from different individual of the *Tapiscia sinensis* trees growing in the Qinling Mountains, Shaanxi province, China (E108°35’, N33°31’). Light blue indicates there were variation, cyan indicated few locus can not align with the others, black indicates that there were high similarity. Red rectangle indicates the primer location and sequence.


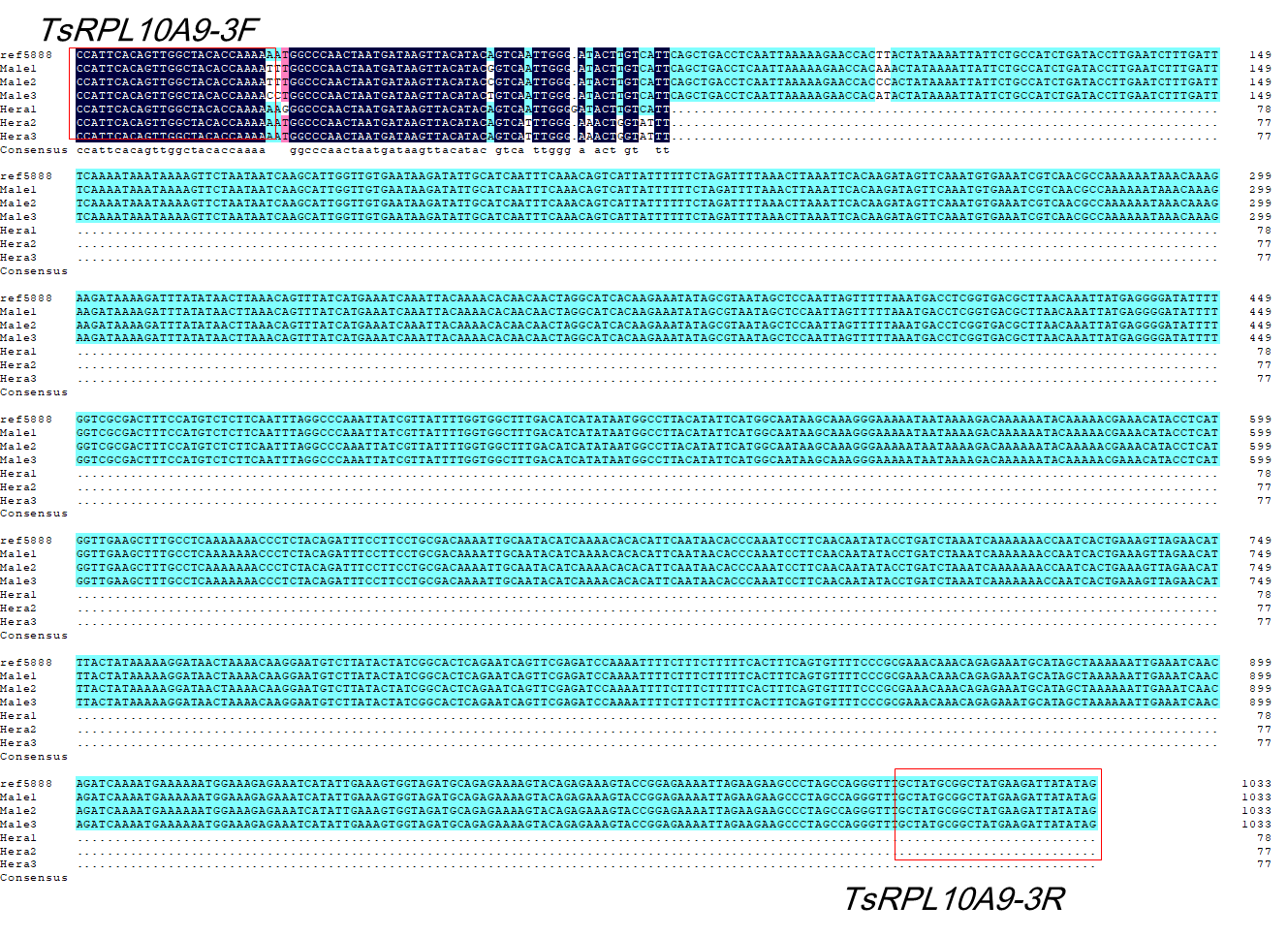


Supplementary Fig. 11. The third PCR Sequence alignment of male and hermaphroditic (Hera) trees at RPL10A aligned with the reference genome.Primer information details see in Supplementary Table 22. Ref: reference, consensus indicates that the same sequence. The Male 1, 2, 3 and Hera 1, 2, 3 were biological replicates indicates that leaf samples from different individual of the *Tapiscia sinensis* trees growing in the Qinling Mountains, Shaanxi province, China (E108°35’, N33°31’). Light blue indicates there were variation, cyan indicated few locus can not align with the others, black indicates that there were high similarity. Red rectangle indicates the primer location and sequence.


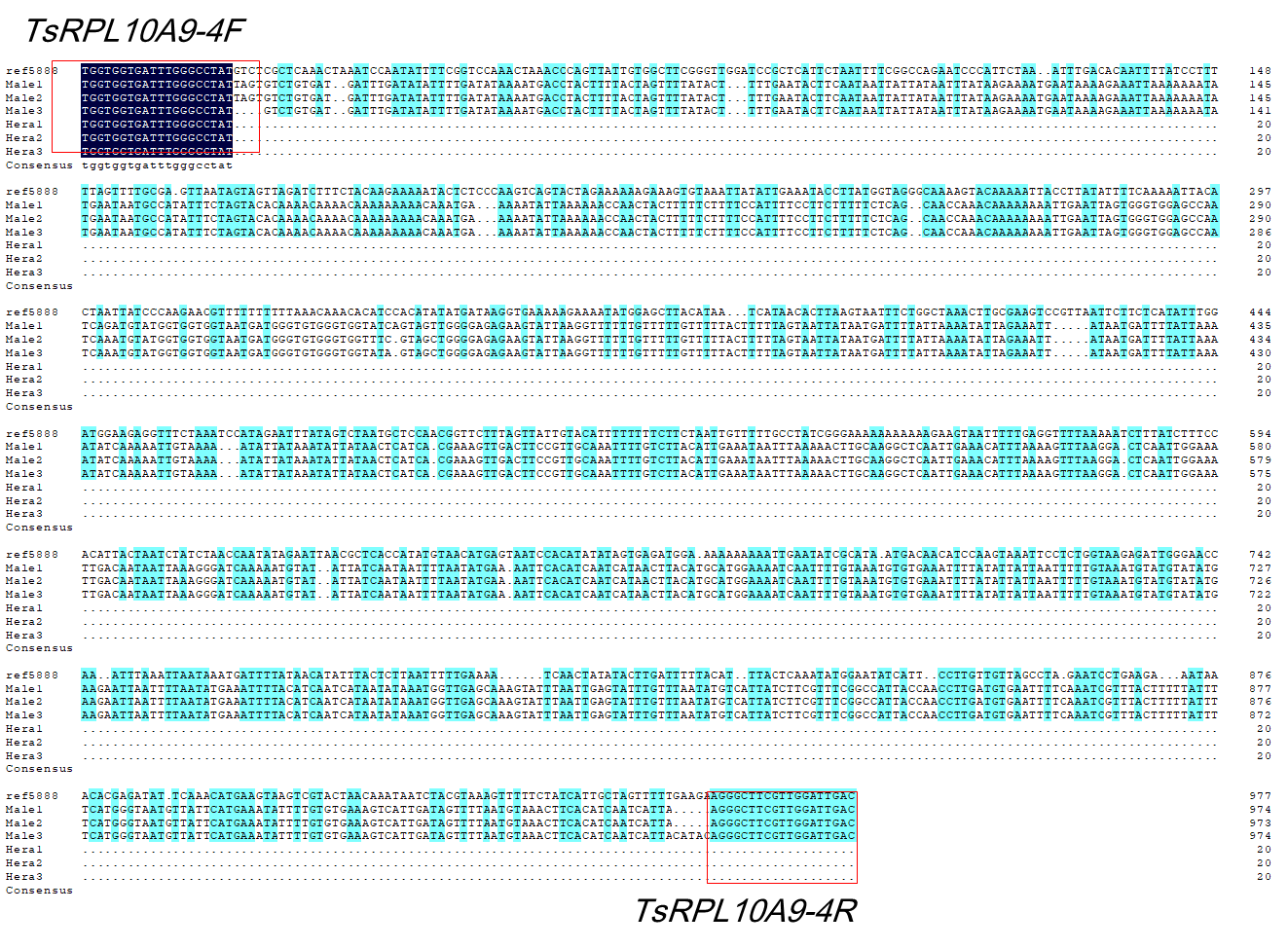


Supplementary Fig. 12. The forth PCR Sequence alignment of male and hermaphroditic (Hera) trees at RPL10A aligned with the reference genome.Primer information details see in Supplementary Table 22. Ref: reference, consensus indicates that the same sequence. The Male 1, 2, 3 and Hera 1, 2, 3 were biological replicates indicates that leaf samples from different individual of the *Tapiscia sinensis* trees growing in the Qinling Mountains, Shaanxi province, China (E108°35’, N33°31’). Light blue indicates there were variation, cyan indicated few locus can not align with the others, black indicates that there were high similarity. Red rectangle indicates the primer location and sequence.


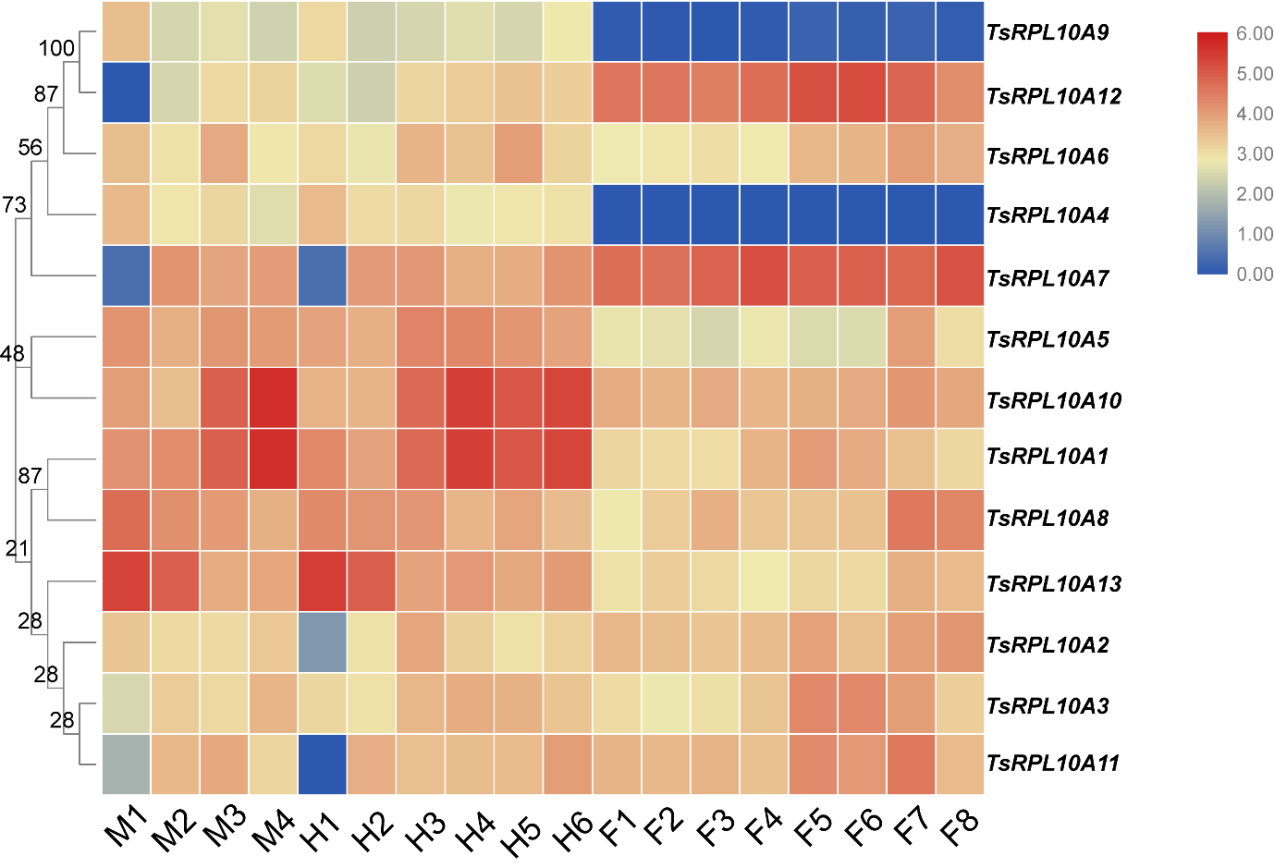


**Supplementary Fig. 13.** Expression of *RPL10A* genes in *T. sinensis* fruit (F), male flowers (M), and hermaphroditic flowers (H). For details concerning the *RPL10* genes, see Supplementary Table 24, for details related to transcriptome sequencing (RNA-Seq) and tissue samples, see Supplementary Table 25.The NJ (neighbor-joining) tree of *RPL10A* gene family members on the left of the figure.


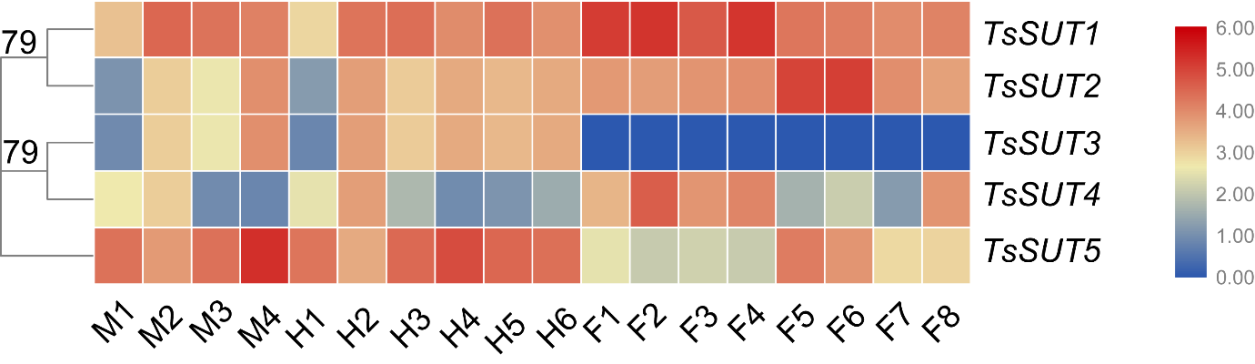


**Supplementary Fig. 14.** Expression of *SUT* genes in *T. sinensis* fruit (F1–F8), male flowers (M1–M4), and hermaphroditic flowers (H1–H6). Details concerning the *SUT* genes are in Supplementary Table 24, and details concerning the transcriptome sequencing (RNA-Seq) and tissue samples are in Supplementary Table 25. The NJ(neighbor-joining) tree of *SUT* gene family members on the left of the figure.


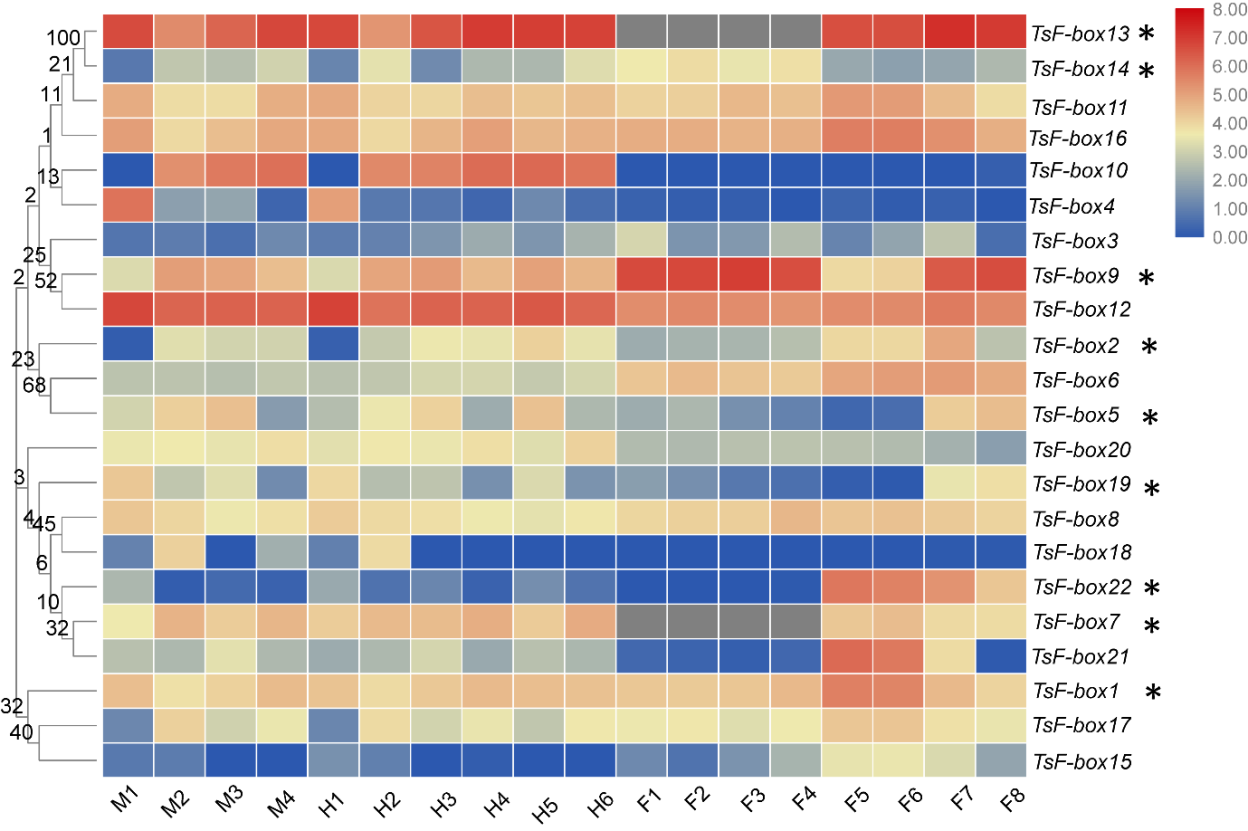


**Supplementary Fig. 15.** Expression of *F-box* genes in *T. sinensis* fruit (F1–F8), male flowers (M1–M4), and hermaphroditic flowers (H1–H6). For details concerning the *F-box* genes see Supplementary Table 24, for details concerning the transcriptome sequencing (RNA-Seq) and tissue samples, see Supplementary Table 25. The *TsF-box* genes indicated with * contain a self-incompatibility (*S-locus*) conserved domain. The NJ(neighbor-joining) tree of *F-box* gene family members on the left of the figure.


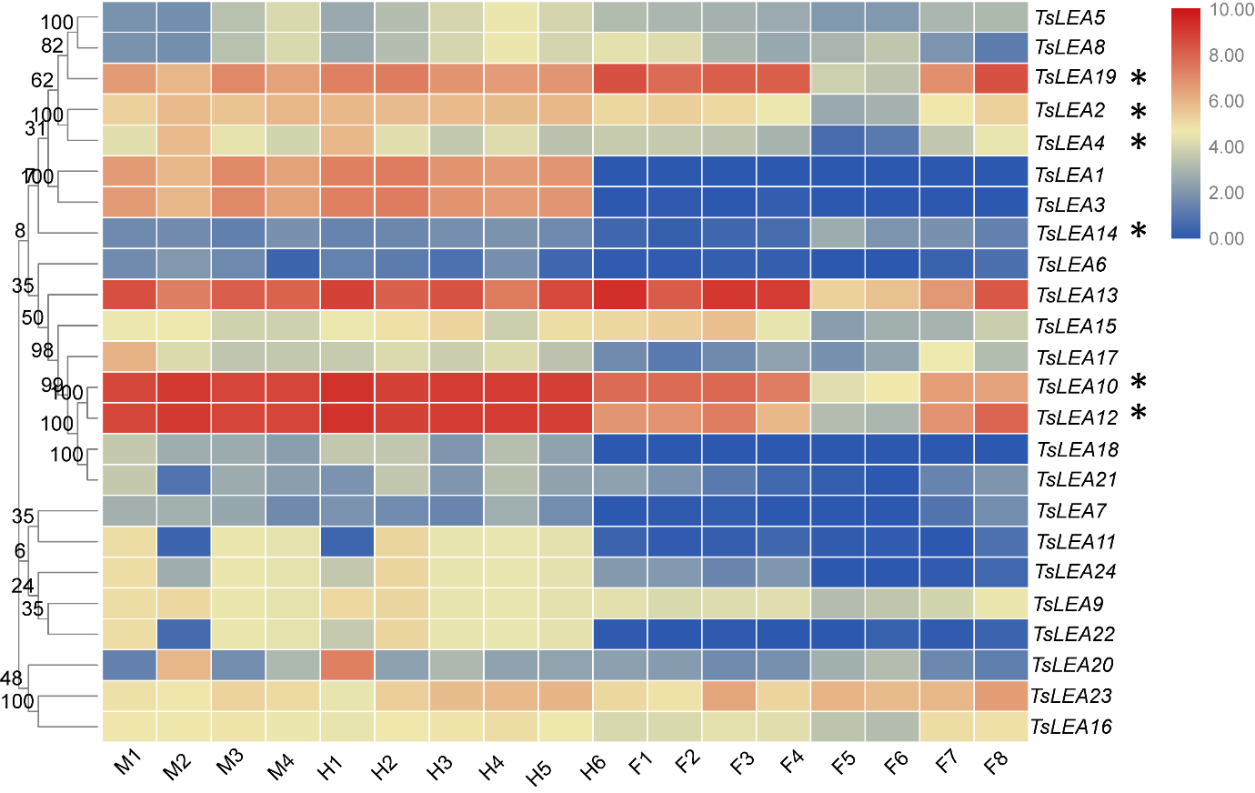


**Supplementary Fig. 16.** Expression of *LEA* genes in *T. sinensis* male flowers (M), hermaphroditic flowers (H) and fruit (F). Descriptions of the *LEA* genes are in Supplementary Table 24, and details related to the tissue samples used for transcriptome sequencing (RNA-Seq) are in Supplementary Table 25. The *TsF-LEA* genes indicated with 6 genes were expressed at significantly different levels in dormant versus developing fruit (Fig. 5c). The NJ (neighbor-joining) tree of *LEA* gene family members on the left of the figure.


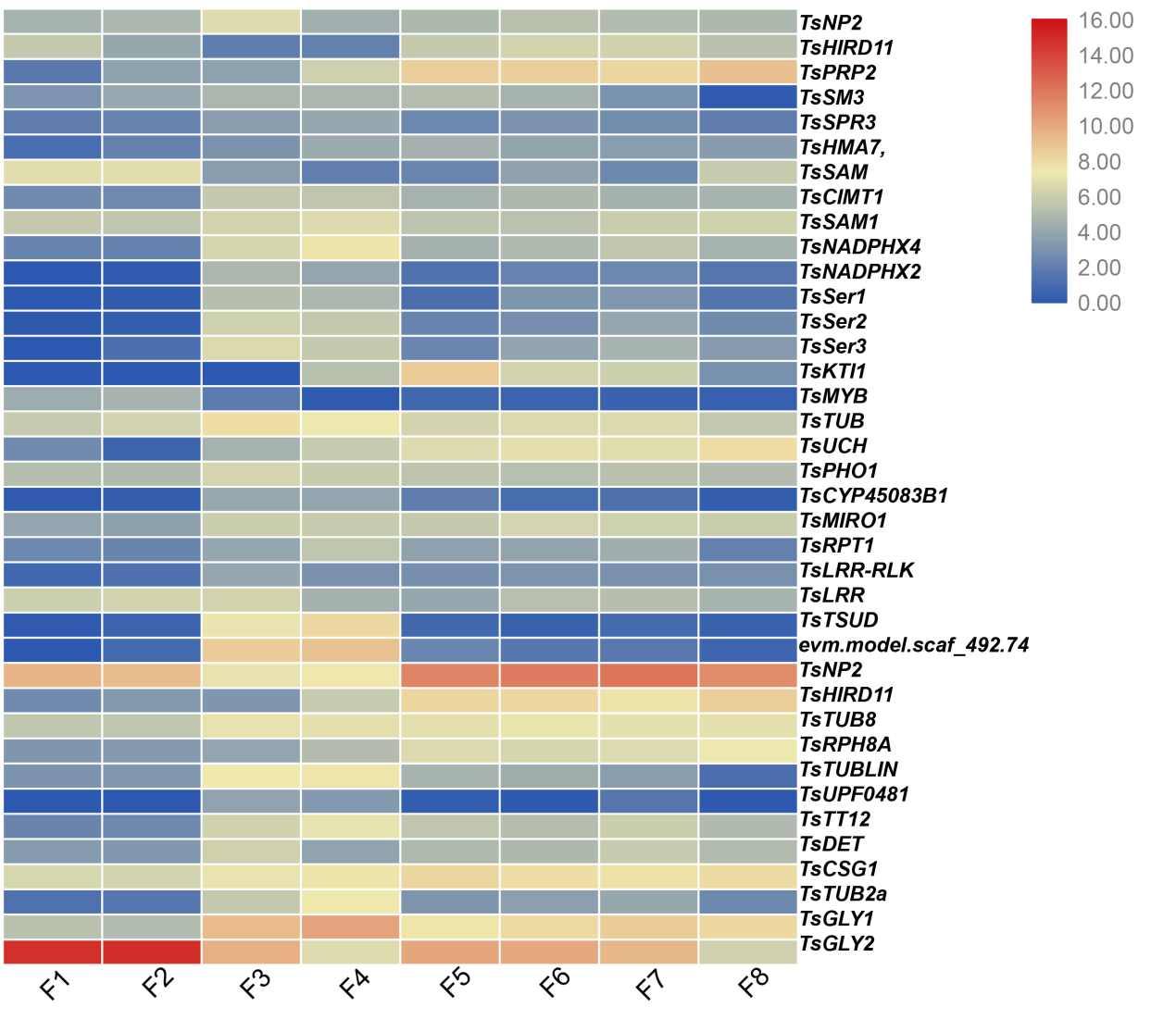


**Supplementary Fig. 17.** Expression of 39 selected genes in *T. sinensis* fruit at eight stages of dormancy and development. For details concerning the 39 selected genes, see Supplementary Table 26.
